# Recent advances in single fluorescent probes for monitoring dual organelles in two channels

**DOI:** 10.1002/smo.20240040

**Published:** 2024-12-15

**Authors:** Xiu‐Zhi Yang, Shankun Yao, Jisong Wu, Jiajie Diao, Weijiang He, Zijian Guo, Yuncong Chen

**Affiliations:** ^1^ State Key Laboratory of Coordination Chemistry School of Chemistry and Chemical Engineering Chemistry and Biomedicine Innovation Center (ChemBIC), ChemBioMed Interdisciplinary Research Center Nanjing University Nanjing China; ^2^ Department of Cancer Biology University of Cincinnati College of Medicine Cincinnati Ohio USA; ^3^ Nanchuang (Jiangsu) Institute of Chemistry and Health Nanjing China; ^4^ Department of Cardiothoracic Surgery Nanjing Drum Tower Hospital Medical School Nanjing University Nanjing Jiangsu China

**Keywords:** bioimaging, dual‐color, dual‐targeted, fluorescent probes, organelle interaction

## Abstract

Organelles are specialized areas where cells perform specific processes necessary for life and actively communicate with each other to keep the whole cell functioning. Disorders of the organelle networks are associated with multiple pathological processes. However, clearly and intuitively visualizing the highly dynamic interactions between ultrafine organelles is challenging. Fluorescence imaging technology provides opportunities due to the distinct advantages of facile, non‐invasiveness and dynamic detection, making it particularly well‐suited for applications in uncovering the mysterious veil of organelle interactions. Regrettably, the lack of ideal fluorescence agents has always been an obstacle in imaging the intricate behaviors of organelles. In this review, we provide a systematic discussion on the existing dual‐color and dual‐targetable molecular sensors used in monitoring organelle interactions, with a specific focus on their targeting strategies, imaging mechanisms and biological applications. Additionally, the current limitations and future development directions of dual‐targetable probes and dual‐emissives are briefly discussed. This review aims to provide guidance for researchers to develop more improved probes for studying organelle interactions in the biomedical field.

## Graphical abstract

This review systematically summarizes the research progress of single fluorescent probes which can direct target organelle interaction networks and images in a two‐color manner, with an emphasis on their dual‐organelle labeling strategies, color conversion mechanisms and biological applications. Meanwhile, the current limitations and a perspective in this area are also briefly discussed.

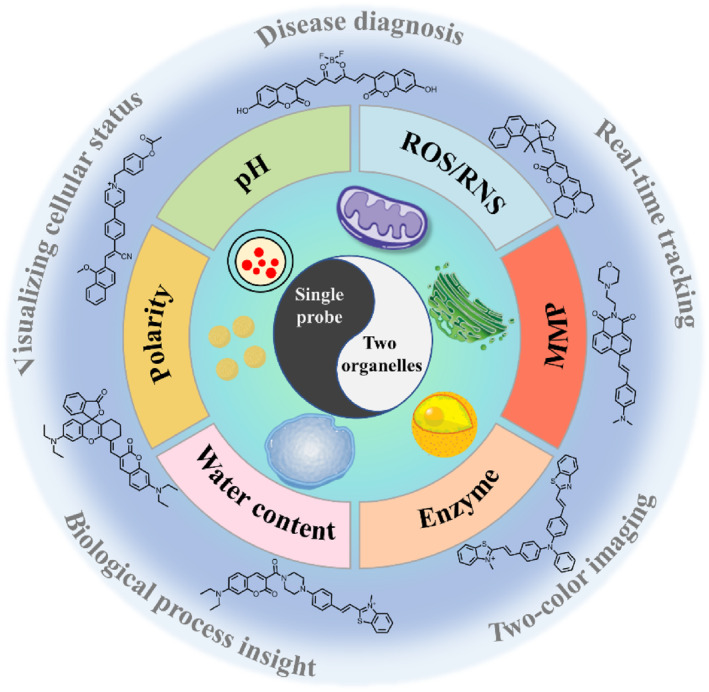

## 1 INTRODUCTION

In order to better maintain the orderly operation of the cellular life system, eukaryotic cells have evolved different subcellular compartments, such as cell membranes, endoplasmic reticulum (ER), lipid droplets (LDs), mitochondria, lysosomes, nucleus, peroxisome, Golgi apparatus, etc.[^1^, ^2^, ^3^] Each organelle performs unique physiological functions since its distinctive morphological structure, composition and microenvironment.[^4^, ^5^, ^6^, ^7^] In addition to participate independently, organelles can work synergistically to form interaction networks to regulate crucial physiological processes.[^8^, ^9^] Organelles interactions occur mainly at the membrane contact sites (MCSs), in complex biological events that help the cell regulate energy metabolism, information transmission and material exchange, thereby maintaining cellular homeostasis and influencing survival processes.[^10^, ^11^] For example, LDs continuously interplay with mitochondria can regulate lipid metabolism.[^12^, ^13^] Mitochondria‐ER communication plays a significant role in orchestrating cellular Ca^2+^ homeostasis.[^14^, ^15^, ^16^] Lysosomes can sense nutrient availability and maintain cellular homeostasis by digesting biological polymers and various dysfunctional organelles.[^17^, ^18^] Accordingly, any impair to the network of organelle interactions can potentially result in malfunctions and diseases occurrence.[^19^, ^20^] Therefore, simultaneous visualization of multiple organelles in the dynamic cellular events has become an important research area.

Electron microscopy (EM) techniques are often utilized to indicate the position and morphology of organelles benefiting from their high resolution even at the nanometer scale.[^21^, ^22^] However, EM is a time‐consuming process and unsuitable for revealing the dynamic changes of organelles in living cells. Fluorescence imaging technology based on organic small molecular dyes is indispensable in biological research owing to its capability to provide dynamic and real‐time information of organelles in a non‐invasive manner.[^23^, ^24^, ^25^, ^26^, ^27^, ^28^] Traditional fluorescence‐based strategies used to visualize organelle interactions are co‐staining two probes targeting different organelles. However, asynchronous imaging due to different uptake rates and increased cytotoxicity in comparison with staining one probe restrict their further practical application. Using single fluorescent probes that targeting two organelles for studying organelle interactions is more attractive and challenging than using two probes that target only one organelle. This requires the probe to meet two requirements simultaneously: (1) it has dual‐labeled capabilities accumulated in two or more organelles; (2) it can provide distinct fluorescence signals without mutual interference in these organelles. The development of dual‐targeted and dual‐emissive single fluorescent probes is still in its infancy, and achieving such molecules still requires trial and error. Three years ago, Lin and co‐workers reported a review article on the fluorescent probes that can target duple organelles.^[29]^ In 2024, Zhou et al. reviewed the single‐molecule to light up the interaction network between LDs and other organelles.^[30]^ Recently, interest in developing single‐probes capable of dual‐color imaging of two organelles concurrently has sharply increased. In order to better investigate the organelle interplay networks and provide guidance for researchers to develop this type of special and significant probes, we systematically summarized the latest results in this field (2017–2024).

**FIGURE 1:**
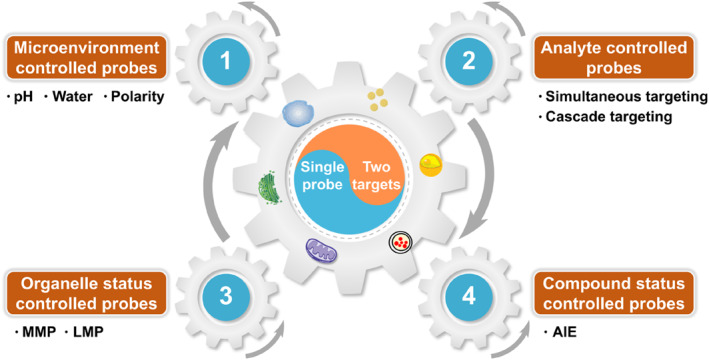
Schematic diagram of the design strategies of dual‐targeted fluorescent probes.

In this review, we have summarized and discussed the dual‐targeted fluorescent probes capable of simultaneous and discriminative visualization of the interaction between two organelles in diverse emission manners (Figure 1). To intuitively grasp the structure–performance relationship, we classified them into four categories according to their imaging mechanism: microenvironment controlled fluorescent probes, analyte controlled fluorescent probes, organelle status controlled fluorescent probes and compound status controlled fluorescent probes. Each of categories, special emphasis is placed on the structural characteristics, working principles and biological applications of each probe. Now we stand at the beginning of a long journey with challenges and opportunities ahead. We hope this review will help researchers gain inspiration to create more excellent probes to explore the mechanism of organelle interactions, elucidate their relationship with the pathogenesis of diseases and enrich tools for human disease diagnosis.

## 2 GENERAL DESIGN STRATEGIES OF ORGANELLE‐ANCHORED FLUORESCENT PROBES

**FIGURE 2:**
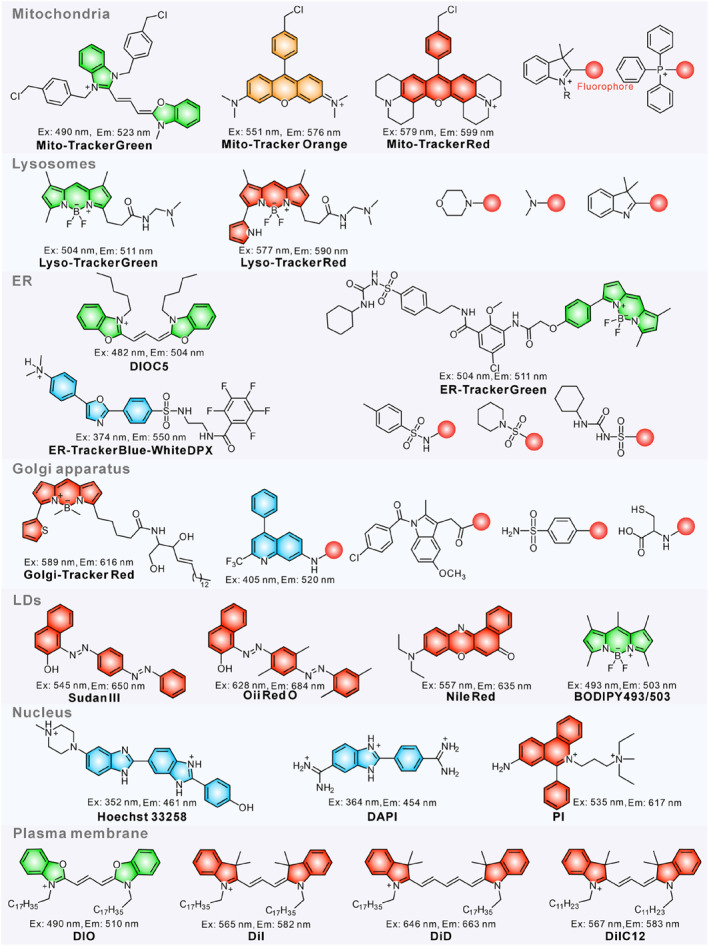
Structures of commercialized organelle‐targeted fluorescent probes and common groups for targeting diverse organelles.

In principle, single chromophore modification with two organelle anchors may attain the dual‐organelle targeting capability.[^29^, ^30^, ^31^] One apparent and important reality is that striking a balance between these two affinities is necessary for targeting duple organelles simultaneously. In addition, by utilizing PET, ICT and FRET mechanisms, the fluorescence characteristics of probes can be regulated to exhibit different fluorescence signals in specific organelles, and single‐molecules capable of discriminating different organelles are achieved. Herein, we concisely discuss the general characteristics of organelles and their respective targeting groups (Figure 2).

Mitochondria, the principal energy centres of living cells, displaying a strong negative transmembrane potential (ΔΨ_m_) at approximately 180 mV within the matrix due to the proton pumping effect induced by oxidative phosphorylation.[^32^, ^33^] Mitochondrial membrane potential (MMP) facilitates the lipophilic cationic dye to penetrate plasma membrane and target mitochondria efficiently by electrostatic force, such as the commercial Mito‐Tracker dyes (rhodamine and cyanine derivatives).^[34]^ Many positively charged units including triphenylphosphonium (TPP),[^35^, ^36^] pyridinium,[^37^, ^38^, ^39^] quinolinium and indolium have been widely used as targeting groups for mitochondria.[^40^, ^41^, ^42^, ^43^]

Lysosome is a single‐layer membrane saclike organelle, with a diameter of about 0.2–0.8 μm, mainly responsible for degradation of macromolecules using digestive enzymes that function only under acidic conditions.^[44]^ Generally, there are two main methods employed to design lysosomes‐targeted fluorescent probes. One way is to equip the core skeleton with the weakly basic lipophilic amines to target lysosomes by leveraging its acidic matrix (pH = 4.5–6.5).^[45]^ Morpholine and dimethylamino are the primarily employed lysosomes‐anchoring units for lysosomal localization.[^46^, ^47^] Another strategy is based on the lysosome—mediated endocytosis pathway, where probe molecules aggregated into nanoparticles can be transported to cells through endocytosis and ultimately captured by lysosomes.^[48]^

The ER is the largest membranous organelle, located adjacent to the nuclear membrane.^[49]^ The ATP‐sensitive K^+^ channel linked to sulfonylurea receptors is widely distributed in the ER membrane.^[50]^ Glibenclamide and sulfonamide derivatives are commonly employed to design ER‐targetable probes because they can bind specifically to sulfonylurea receptors.[^51^, ^52^] For example, the commercial ER‐Tracker dyes (ER‐Tracker Red and ER‐Tracker Green) are constructed by linking glibenclamine with BODIPY. Meanwhile, ER contains the largest membrane surface area, which has high lipid solubility and certain membrane potential. Some lipophilic cationic molecules tend to localize in the ER according to the principle of “like dissolves like” and electrostatic force. For instance, the first ER‐targeted probes (DiOC_5_ and DiOC_6_) were constructed mainly based on the mechanism.^[53]^

The Golgi apparatus is mainly responsible for the transportation and secretion of proteins and enzymes.^[54]^ Golgi localization groups can be mainly divided into three classes. Some 7‐aminoquinoline derivatives with a trifluoromethyl moiety can specifically target Golgi apparatus.^[55]^ The commercially available Golgi‐targeting probes Golgi‐Tracker Green and Golgi‐Tracker Red and other fluorophores incorporated with L‐cysteine group localized to the Golgi by specific binding to cysteine residues or cysteine in the Golgi apparatus.^[53]^ Besides, the phenylsulfonamide moiety and inhibitor derivatives of cyclooxygenase‐2 (e.g., indomethacin and celecoxib) target the Golgi apparatus through embedding into specific pockets of cyclooxygenase‐2, which are overexpressed in the Golgi apparatus.[^56^, ^57^]

Ubiquitous in cells, LDs are central hubs for lipid metabolism and trafficking, containing a neutral lipid core enclosed by a phospholipid monolayer and proteins. Owing to the extreme hydrophobicity in the oil core, organic dyes equipped with a highly lipophilic molecular scaffold are prone to orient in the most apolar structures via the hydrophobic interactions.[^58^, ^59^, ^60^] A typical example is the commercial LDs‐Tracker dyes which are highly lipophilic dyes with high hydrophobic constants.^[61]^

The cell nucleus is the primary organelle for storing hereditary information, which is enclosed by a double‐layer membrane and isolated from the cytosol.^[54]^ The nucleus of eukaryotic cells contains the vast majority of negatively charged DNA and RNA; some planar aromatic cation scaffolds can bind to DNA/RNA in the minor groove by electrostatic interaction, thus realizing nucleus localization.^[62]^ Several of them are already available for purchase, such as Hoechst, DAPI and Propidium Iodide (PI).

The plasma membrane is the first defensive barrier of cells, which is critical for providing a stable environment for internal organelles and maintaining cellular integrity and function.[^63^, ^64^] Based on its phospholipid bilayer structure, a typical strategy in designing membrane‐anchored probes involves grafting long hydrophobic alkyl chains or positively charged ammonium units onto fluorophores via hydrophobic and electrostatic interactions with phospholipid bilayers.[^65^, ^66^, ^67^, ^68^, ^69^] Notably, some commercial dyes achieve membrane‐anchoring specificity by introducing long hydrophobic chains.

## 3 MICROENVIRONMENT CONTROLLED FLUORESCENT PROBES

Intracellular organelles are maintained in a specific microenvironment appropriate to perform their particular cellular functions, ratiometric environment‐sensitive dyes show potential applications in realizing the two‐color discrimination of double organelles theoretically. If such probes are distributed simultaneously in two organelles, the microenvironment differences may enable them to exhibit emission wavelength shift in the two organelles. Compared with the single‐targeted probes, the multifunctional probes can present fluctuations in the cellular microenvironment and synergistic interactions of multiple organelles in a better and clearer way.

### 3.1 Water‐sensitive dual‐targeted fluorescent probes

**FIGURE 3:**
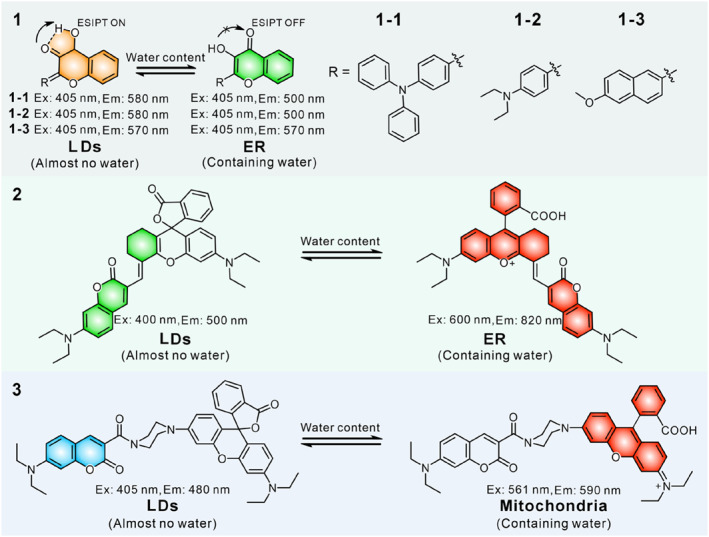
The molecular structures of water‐sensitive dual‐targeted fluorescent probes.

**FIGURE 4:**
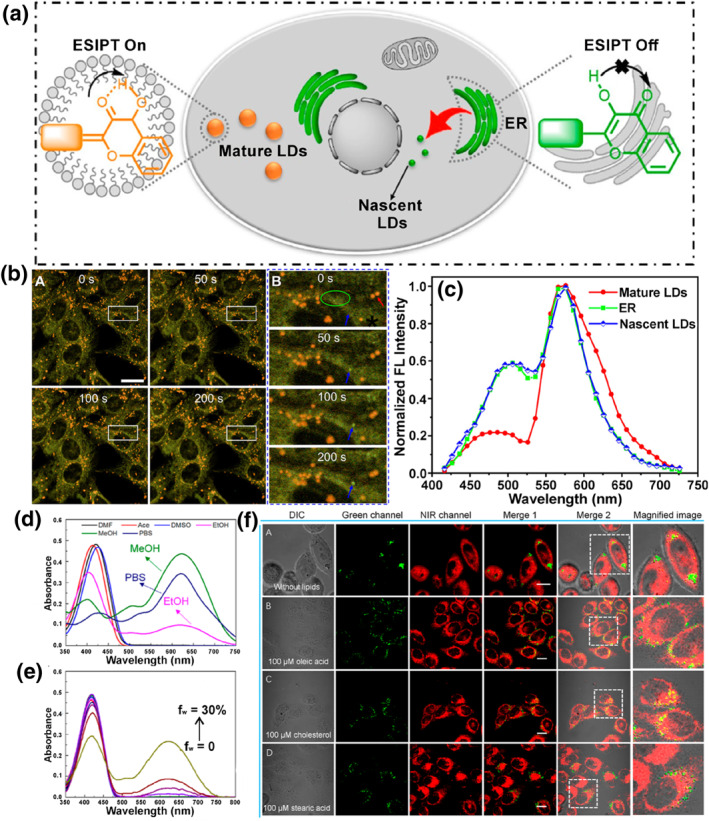
(a) Schematic Illustration of probe **1** and the dual‐color visualization mechanism of LDs and ER. (b) Real‐time dynamic tracking LD formation in HeLa cells induced by oleic acid using probe **1–1**. (c) In situ fluorescence spectra of mature LDs, nascent LDs, and ER. (d) Absorption spectra of probe **3** in various solvents. (e) Absorption spectra of probe 3 in different ratios of water and acetone. (f) Fluorescence imaging of probe **3** under different conditions. Reproduced with permission from refs. [^70^, ^73^] Copyright 2021 American Chemical Society.

LDs are monolayer membrane organelles filled with various neutral lipids, which are extremely hydrophobic organelles. Apart from the LDs, the vast majority of organelles are bilayer membrane structures containing some aqueous component.^[29]^ The variations in water fraction provide an approach for the design of dual‐targeted single‐probes for LDs and other organelles. As shown in Figure 3, we list the single fluorescent probes that could target two organelles in a dual‐color manner due to differences in organelle water content. Yu's group constructed three lipophilic water‐sensitive probes **1** (**1–1**, **1–2** and **1–3**), which could visualize and differentiate both LDs and ER simultaneously in dual channels for the first time (Figure 4a–c).^[70]^ These probes were designed by introducing different electron‐donating groups into the 3‐hydroxyflavone skeleton, which is a classical excited state intramolecular proton transfer (ESIPT) molecule. The appropriate lipophilicity enabled the probes to stain both LDs and the ER. Additionally, probes (**1–1** and **1–2**) with strong electronic donor exhibited highly sensitive fluorescence responses to the subtle differences in water contents between LDs and ER membrane, labelling LDs and ER in orange and green emission colors in the lambda mode, respectively. By utilizing probes (**1–1** and **1–2**), the morphology, volume, number and distribution of LDs and ER in various cells and tissues have been clearly recorded, and reinforced the prevailing hypothesis that LDs may derive in the ER.

Rhodamine dye is a privileged scaffold in fluorescent probe design owing to its tunable photophysical properties. Rhodamine derivatives are sensitive to changes in the water content; in aprotic solvents, they exist in a ring‐closed form, while in protic solvents, they exist in a ring‐opened form.^[71]^ In 2022, Tian and co‐workers designed probe **2** by directly linking a coumarin moiety to a rhodamine dye.^[72]^ The rhodamine part underwent intramolecular spirocyclization reaction in hydrophobic LDs to illuminate a green emission (*λ*_em, max_ = 500 nm), while existing in a ring‐opened form in the ER to emit red fluorescence (*λ*_em, max_ = 820 nm), the crosstalk between the two channels could be ignored (up to 320 nm). With the probe, the changes in the number, size and distribution of LDs and the ER in living cells and various tissues under different treatments were revealed. It was found that lipid consumption was accelerated at both high temperature, low temperature and hypoxic stimulation, while lipid accumulation occurred under high lipid treatment. There were dramatic differences in the amount of ER and LDs among different tissues, where white adipose tissue had the highest LDs amount and the lowest ER amount compared to lung tissue, liver tissue and myocardium.

In 2021, Lin and co‐workers constructed the first fluorescent probe **3** to separately visualize LDs and mitochondria in dual channels based on the reversible spirocyclic reaction of the rhodamine dye (Figure 4d–f).^[73]^ The probe also existed in two forms: the neutral lipophilic ring‐closed form and the cationic ring‐opened form. The cationic ring‐opened form structure could stain mitochondria displaying negative membrane potential to give red fluorescence (*λ*_em, max_ = 590 nm), while the neutral lipophilic ring‐closed form structure accumulated on hydrophobic LDs with blue color (*λ*_em, max_ = 480 nm). Based on the difference in water fraction between LDs and mitochondria inner membrane, LDs and mitochondria could be clearly visualized in blue and red channels in living cells. With the probe, the authors successfully monitored increased LDs‐mitochondria contact in nutrient deficient and lipid rich conditions. In addition, analysis of the distribution of LDs and mitochondria in various tissues revealed frequent LDs‐mitochondria contact in lung and brown adipose tissues.

### 3.2 Polarity‐sensitive dual‐targeted fluorescent probes

**FIGURE 5:**
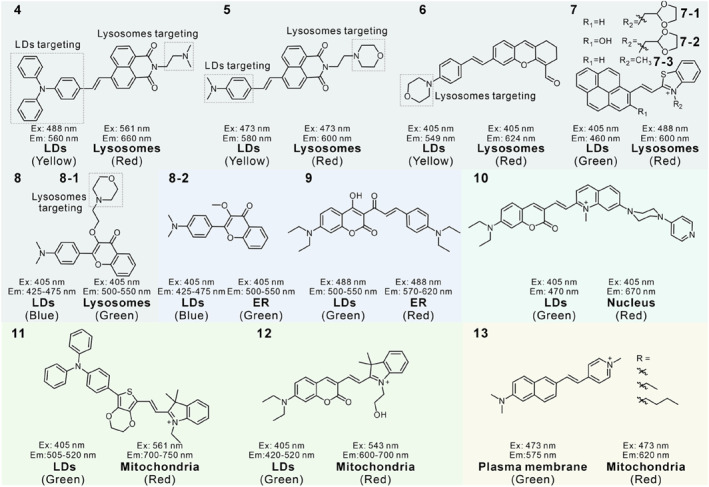
The molecular structures of polarity‐sensitive dual‐targeted fluorescent probes.

Fluorescence dyes with the “donor (D)–π–acceptor (A)” molecular scaffold are susceptible to changes in polarity due to the intramolecular charge transfer (ICT) effect.^[74]^ Typically, when the polarity of the surrounding environment increases, the emission of these solvatochromic dyes will shift to a longer wavelength.^[75]^ LDs and plasma membranes present lower polarity in cells compared with other organelles composed of unsaturated sidechains. Typically, two organelles with different polarities can potentially be distinguished in two colors if they are both stained with such solvatochromic dyes (Figure 5).

Based on this design strategy, in 2019, Yang and co‐workers developed probe **4** by combining triphenylamine (acted as an electronic donor) and 1,8‐naphthalimide (acted as an electron acceptor) via a π‐bridge.^[76]^ The probe with ICT character was sensitive to environmental polarity, displaying two separate emissive peaks in toluene (*λ*_em, max_ = 549 nm) and DMSO (*λ*_em, max_ = 670 nm), respectively. An additional weakly basic dimethylamino group had the ability to protonate which could drive the accumulation of the probe in lysosomes. Meanwhile, the molecule was highly lipophilic and could target the hydrophobic LDs. Taking advantage of the inherent polarity difference between LDs and lysosomes, probe **4** illuminated LDs and lysosomes in yellow and red colors, respectively. This is the first time that a single probe could discriminate lysosomes and LDs in two separate emission colors. The imaging results in different cell lines indicated that HepG2 cells contained LDs with larger size, and TTF cells might have more lysosomes and small‐sized LDs. The authors also used the probe to real‐time track the LDs and lysosomes kinetics in living cells and the distribution of the two organelles in zebrafish embryos.

In 2020, another fluorescent sensor **5** based on 1,8‐naphthalimide derivative was developed by Yu and co‐workers.^[77]^ It could also label LDs and lysosomes simultaneously in live cells and tissues in the dual‐emissive channels. In this architecture, the morpholine moiety was responsible for lysosomes localization because it has an inherent affinity for acidic lysosomes. The dimethylamino moiety acted as an LDs targeting unit due to its lipophilic property. Because of the polarity difference in the LDs core and lysosomal membrane and the D‐π‐A type probe is sensitive to environmental polarity, the dual‐color visualization of LDs (*λ*_em, max_ = 580 nm) and lysosomes (*λ*_em, max_ = 600 nm) in yellow and red emission colors was achieved using the probe in Lambda mode. Utilizing probe **5**, they successfully observed a decrease in the amount of LDs and no significant changes in the lysosomes in glucose‐deprived cells, which indicated that cells would consume lipids stored in LDs to maintain metabolism when suffering from starvation. The dynamic process of lysosomes to remove unwanted LDs in the lipophagy process in living cells was also monitored with the probe. Moreover, the shape, size and distribution of LDs and lysosomes in multiple mice tissues were also studied, the results indicated that LDs and lysosomes were always closely associated and liver tissue had more LDs than other tissues.

In 2022, Bai's group constructed a typical D‐π‐A structure based on the xanthene fluorophore, forming an ICT process, where the aldehyde unit acted as the electron‐withdrawing group and the morpholine ring as the electron donating group and was used for targeting lysosomes.^[78]^ Probe **6** could distinguish LDs and lysosomes in yellow and red emission colors. The results of cell imaging showed that the probe could serve as a powerful imaging tool to track the dynamic evolution of LDs and lysosomes.

Recently, Liu et al. reported a ratiometric polarity‐sensitive fluorescent probe **7–1** for tracking liquid–liquid phase separation (LLPS) and intracellular polarity changes.^[79]^ In the design of the probe, they combined the lipophilic pyrene fluorophore and hydrophilic benzothiazolium salt through the Knoevenagel condensation reaction to form a D‐π‐A configuration. Hydroxyl unit and 1,3‐dioxopentacyclic group were utilized to balance the lipophilicity/hydrophilicity. The probe **7–1** could monitor the environmental polarity changes benefiting from its ICT effects, and the amphiphilic nature allowed it to visualize the phase‐change process via fluorescence color changes. In addition, this probe could illuminate both LDs (green) and lysosomes (red) in separate emission channels. The probe offered the ability to monitor the behaviour of LDs or lysosomes in organelle interactions from both polarity and phase transition perspectives.

In 2021, Tian's group developed two polarity‐sensitive probes **8** (**8–1** and **8–2)** based on the ICT mechanism.^[80]^ Probe **8–1** possessed a weakly basic morpholine ring to label lysosomes, and its lipophilic structure might drive accumulation in the LDs. Probe **8–2**, without a morpholine group, targeted hydrophobic organelles such as LDs and ER due to its lipophilic nature. Probe **8–1** could distinguish spherical structure LDs and lysosomes in blue and green emission colors, and probe **8–2** enabled discrimination of LDs and ER in blue and green emission colors, respectively. By virtue of the two probes, the number of LDs and the dynamics interaction of LDs‐Lysosomes and LDs‐ER under different stimuli was clearly revealed.

In 2023, Kong's group designed probe **9** with a distinct D‐π‐A‐π‐D framework to further study the detailed interactions between LDs and ER.^[81]^ Probe **9** displayed red‐shifted emissions with the increment in polarity caused by the ICT process. The probe could image LDs and ER with green and red fluorescence separately. The dynamic behaviors of LDs and ER under starvation and oleic acid stimulations were clearly monitored by probe **9**.

**FIGURE 6:**
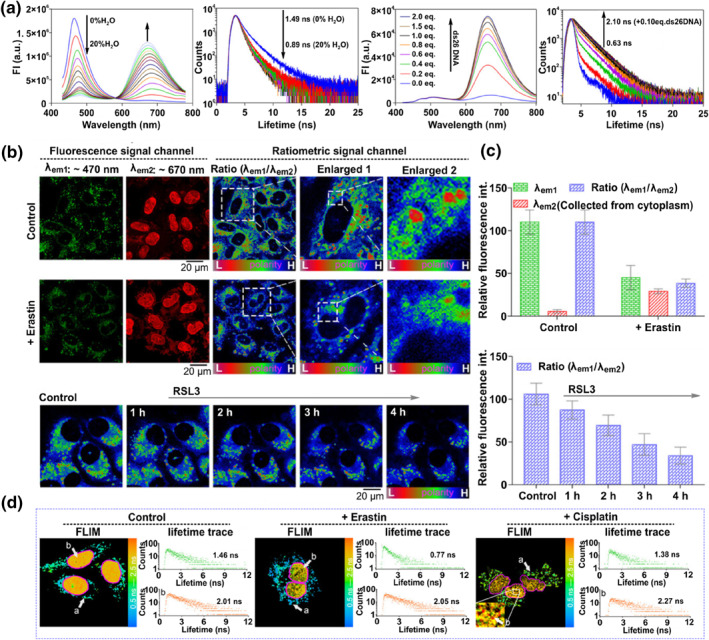
(a) Fluorescence and fluorescence lifetime spectra of probe **10** response to polarity and ds26DNA. (b) Biological application of probe **10** in visualizing polarity change in Erastin or RSL3 induced ferroptosis. (c) The relative fluorescence intensity in the fluorescence images (b). (d) Application of probe **10** to monitor changes in LDs and nuclear polarity under diverse stimuli through FLIM. Reproduced with permission from ref.^[82]^ Copyright 2021 Wiley‐VCH.

The difference between the nucleus and the LDs provides a design strategy for imaging the two organelles in dual‐color. In 2021, probe **10** was constructed by optimizing the lipophilicity and nucleic acid binding ability, enabling it to accumulate simultaneously in LDs and nuclei, which is the first case for dual‐color and dual‐targeted LDs and nuclei by a single probe.^[82]^ The probe consists of three parts: the lipophilic coumarin unit targeted LDs and acted as an electronic donor, cationic quinolinium could label electronegative nucleic acids through electrostatic interaction and act as an electronic acceptor, piperazine unit was added to enhance the nucleic acid binding capacity by mimicking the Hoechst 33342 (a commercial nuclear dye). Probe **10** with a push–pull structure showed green fluorescence in the LDs and red fluorescence in the nucleus. The probe could not only track the occurrence and progression of ferroptosis but also clearly observe the increase in LDs polarity during the ferroptosis process via both fluorescence and fluorescence lifetime imaging (FLIM) (Figure 6). The capacity to dual‐color image LD and nuclear could help researchers more effectively study the ferroptosis and related biological processes.

In eukaryotic cells, mitochondria present negative MMP, typically fluctuating from −150 to −180 mV. Thus, membrane permeable organic cations are prone to enrich in mitochondria through the electrostatic interaction. LDs are highly neutral lipophilic environments, and neutral charge molecules with high lipophilicity prefer to locate in LDs according to the theory of similarity and intermiscibility. Owing to the phenomenon of polarity difference between LDs and mitochondria, Zhou's team designed a polarity‐sensitive fluorescent probe **11** based on an electron acceptor indole salt and an electron donating triphenylamine group, 3,4‐ethylene dioxythiophene acted as the *π* bridges.^[83]^ The probe showed green emission (*λ*_em, max_ = 500 nm) in the low‐polarity solvent 1,4‐dioxane and red emission (*λ*_em, max_ = 800 nm) in the high‐polarity solvent DMSO due to the evident ICT effect. Probe **11** with its lipophilicity and cationic salt anchored on mitochondria via electrostatic interaction and targeted LDs with strong hydrophobicity, emitting distinct fluorescence in LDs and mitochondria due to the lower internal polarity of LDs compared to mitochondria. In addition to simultaneously monitoring the changes in quantity and size of LDs and mitochondria in living samples under stimulation, probe **11** also served as a fluorescence tool for early diagnosis of disorders linked to LDs aggregation such as fatty liver.

Taking inspiration from the electric charge discrepancy between mitochondria and LDs, Lin and co‐workers proposed a dual‐emissive probe **12** for simultaneous imaging of mitochondria and LDs.^[84]^ The coumarin‐indolenine fluorophore was designed based on the reversible cyclization and ring‐opening transition, which converted its molecules between the cationic salt form (*λ*_em, max_ = 670 nm) and neutral lipophilic structure (*λ*_em, max_ = 480 nm) with changes in environmental polarity. The mitochondrial polarity is higher than the LDs polarity; therefore, the probe could simultaneously accumulate in mitochondria and LDs and emit in red and green channels, respectively. With the dual‐site/dual‐color probe, the mitochondria‐LDs interactions were thoroughly studied in mitochondrial disorder, LDs accumulation, apoptosis, and low temperature. This series of experiments indicated that mitochondria exert crucial functions in LDs accumulation; the amount and dimension increased during mitochondrial dysfunction due to the delayed consumption of excess liposomes. Meanwhile, LDs played a critical role in assisting mitochondria in coordinating energy supply in fighting the cold.

Generally, plasma membranes containing more saturated phospholipid chains exhibit lower polarity than the organelle membranes composed mostly of unsaturated side chains. Based on this, Yu and colleagues designed a polarity‐responsive probe **13** to simultaneously visualize plasma membranes and mitochondria in two colors.^[85]^ The probe with suitable membrane permeability could stain the plasma membrane (*λ*_em, max_ = 575 nm) and mitochondria (*λ*_em, max_ = 620 nm) separately in green and red colors at normal cholesterol (CL) levels in living cells using Lambda‐based imaging mode. Because the increase and decrease of CL flux altered the membrane permeability and the distribution of the probe, probe **13** could selectively image plasma membranes in yellow fluorescence at increased CL amount, and only displayed red fluorescence in mitochondria when CL amount decreased. Consequently, the CL fluctuation in plasma membranes was tracked via the fluorescence colors and localizations of the spatial controllable‐distribution probe.

### 3.3 pH‐sensitive dual‐targeted fluorescent probes

**FIGURE 7:**
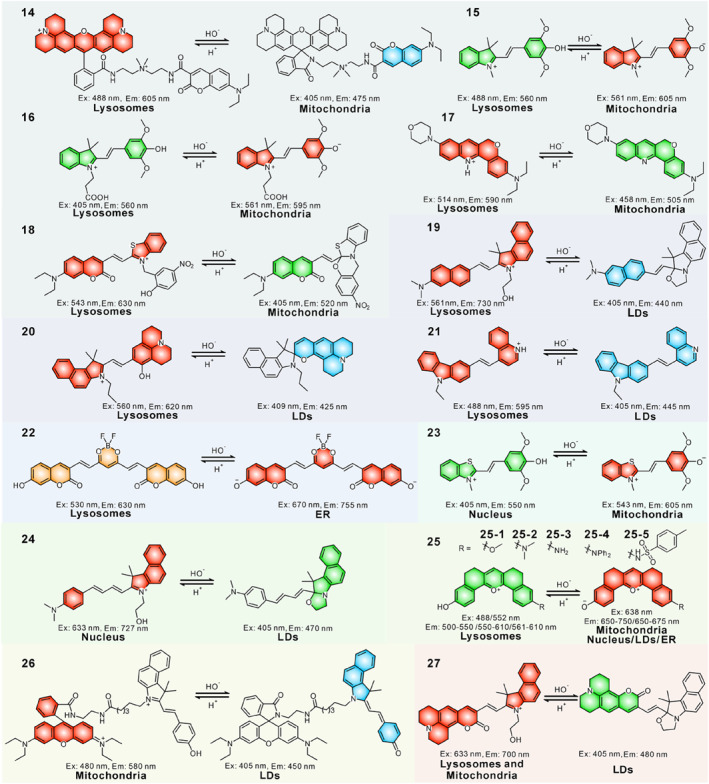
The molecular structures of pH‐sensitive dual‐targeted fluorescent probes.

Apart from water content and polarity, acidic–basic status (pH) also exhibits significant heterogeneity in varying cellular regions. For instance, the pH in lysosomes is just 4.5–6.5, in the ER is about 7.2, while the mitochondrial pH is approximately 8.0.[^86^, ^87^] The pH difference provides an opportunity to design single‐molecule dual‐emissive probes to achieve two‐color differentiation of acidic organelles from others (Figure 7).

In 2017, Han’s group synthesized a dual‐organelle sensor **14**, where the blue emissive coumarin moiety and the acidity‐activatable rhodamine fluorophore were linked through ΔΨ_m_‐responsive quaternary ammonium ion (AMI) linker.^[88]^ When the pH was changed from pH 8.5 to 4.0, the probe exhibited a significant enhancement in fluorescence emission at 605 nm and a slightly decrease at 475 nm. Probe **14** anchored in lysosomes and mitochondria driven by the lysosomal acidity and the negative mitochondrial potential. It showed red fluorescence from the ring‐opened rhodamine part in acidic lysosomes and blue fluorescence from the coumarin moiety in weakly alkaline mitochondria owing to its pH‐sensitive property. The decrease in ΔΨ_m_ drove more probe molecules to migrate from mitochondria to lysosomes benefitting by the lower affinity of AMI for mitochondria compared to TPP (a typical ΔΨ_m_‐responsive entity), causing an increase in red fluorescence and a decrease in blue fluorescence. In contrast, the imbalance in lysosomal acidity led to the molecules escape from lysosomes into mitochondria, and the red fluorescence would attenuate and blue fluorescence would increase. This contribution provided a promising tool to detect the alterations of lysosomal acidity and mitochondrial ΔΨ_m_ via analysing the localization and emission color.

To better discriminatively visualize mitochondrial and lysosomal dysfunctions, a pH‐responsive two‐targeted probe **15** based on a hemicyanine derivative was developed by Tian's group in 2023.^[89]^ In their design strategy, the positively charged indole salt had an affinity to mitochondria and was used to anchor mitochondria, the hydroxyl group was introduced to act as a pH response site, the structure transformed to a weak base form that could label the acidic lysosomes. The ratiometric response to pH fluctuations and the dual‐targeting property enabled it to be red emissive in mitochondria (*λ*_em, max_ = 605 nm) and green emissive in lysosomes (*λ*_em,max_ = 560 nm) concurrently. Experimental results demonstrated that **15** could detect mitochondrial depolarization and lysosomal alkalization via its subcellular immigration and changes in fluorescence intensity of red green channels. Probe **15** targeted mitochondria and lysosomes rely on the negative ΔΨ_m_ and acidic conditions, respectively. During depolarization of ΔΨ_m_, red emission decreased because the probe immigrated from mitochondria into lysosomes; during lysosomal damage, green emission decreased since probe escaped from lysosomes into mitochondria. The authors also applied the probe to visualize mitochondria‐lysosome contacts during autophagy and revealed two regulation patterns.

Subsequently, another pH‐responsive probe **16** based on a similar structure was reported in another paper in the same year by Chen and co‐workers.^[90]^ Probe **16** combined a syringyl derivative on the *N*‐carboxyethyl indolium scaffold with *π* bridge. The cationic indolium moiety targeted the probe to weak alkaline mitochondria and gave a red emission, and the *N*‐carboxyethyl indolium scaffold could also target the probe to acidic lysosomes owing to the lysosomal engulfment and gave a green emission. Taking advantage of the pH‐responsive and dual‐targeted characteristics of probe **16**, the dynamic mitochondria‐lysosome interaction and related pH fluctuations during ferroptosis were clearly tracked. The imaging results showed intensified physical contact between mitochondria and lysosomes and pH disorder in lysosomes during ferroptosis.

Recently, Sun et al. designed a new fluorescent probe **17** to image lysosomes and mitochondria in dual emission colors based on a benzopyranoquinoline skeleton, and morpholine was added to increase the affinity to lysosomes.^[91]^ The probe targeted lysosomes to present in a red‐emissive protonation form, and targeted mitochondria to form a deprotonated structure with green fluorescence. Thus, acidic lysosomes and alkaline mitochondria were imaged with the probe in red (*λ*_em, max_ = 590 nm) and green emission (*λ*_em, max_ = 505 nm) channels, respectively. The ΔΨ_m_ situation and lysosomal alkalization could be detected by observing the localization and emission colors of the probe. The authors employed the probe to distinguish healthy and pathological cells, and observed the dynamic mitochondria‐lysosome physical interaction in autophagy.

**FIGURE 8:**
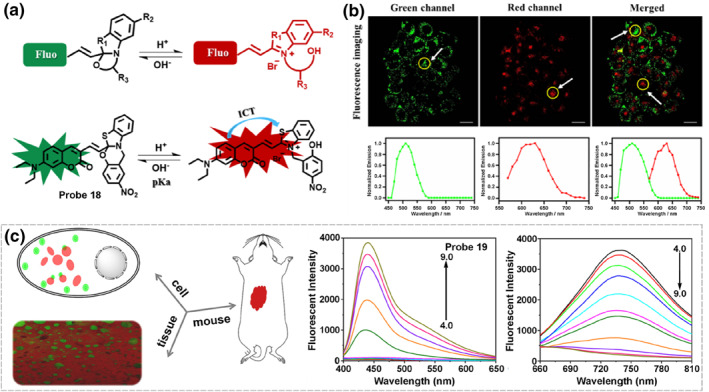
(a) The proposed sensing mechanism of probe 18. (b) Fluorescence imaging and in situ emission spectra of probe 18 in dual channels. (c) Observation of lipophagy under normal and starving conditions utilizing probe 19. Reproduced with permission from refs. [^92^, ^94^] Copyright 2021 American Chemical Society. Copyright 2024 Elsevier.

Compared to lysosomes, neutral LDs display a higher pH and higher hydrophobicity, exploiting the pH difference between lysosomes and LDs provides a possible way to design dual‐targeted and dual‐emissive probes. In 2021, probe **18** was designed to visualize lysosomes and LDs in two emissions utilizing their pH and hydrophilicity differences (Figure 8a,b).^[92]^ It showed distinct photophysical properties through the reversible intramolecular spirocyclization induced by pH. The probe targeted lysosomes in the ring‐open form to give a red emission (*λ*_em, max_ = 630 nm), and formed a neutral ring‐closed skeleton in LDs that emitted green fluorescence (*λ*_em, max_ = 520 nm). The probe was successfully applied to monitor the dynamic lipophagy process.

Similar in molecular structure to probe **18**, probe **19** was also a pH‐dependent hydrophility‐adjustable fluorescent probe, which simultaneously lighted up lysosomes and LDs to yield red (*λ*_em, max_ = 730 nm) and blue emissions (*λ*_em, max_ = 440 nm) for their internal pH differences.^[93]^ The probe was successfully applied to quantify the different regulation in the number of LDs and lysosomes under different stimuli, and was capable of detecting lysosomal pH alteration during the autophagy process. Recently, probe **19** was used to explore LDs‐lysosomes communication during apoptosis and the lipophagy process (Figure 8c).^[94]^ The emergence of probe **19** provided a very promising tool to assist in the timely diagnostic degrees of NAFLD and fatty liver lesions.

**FIGURE 9:**
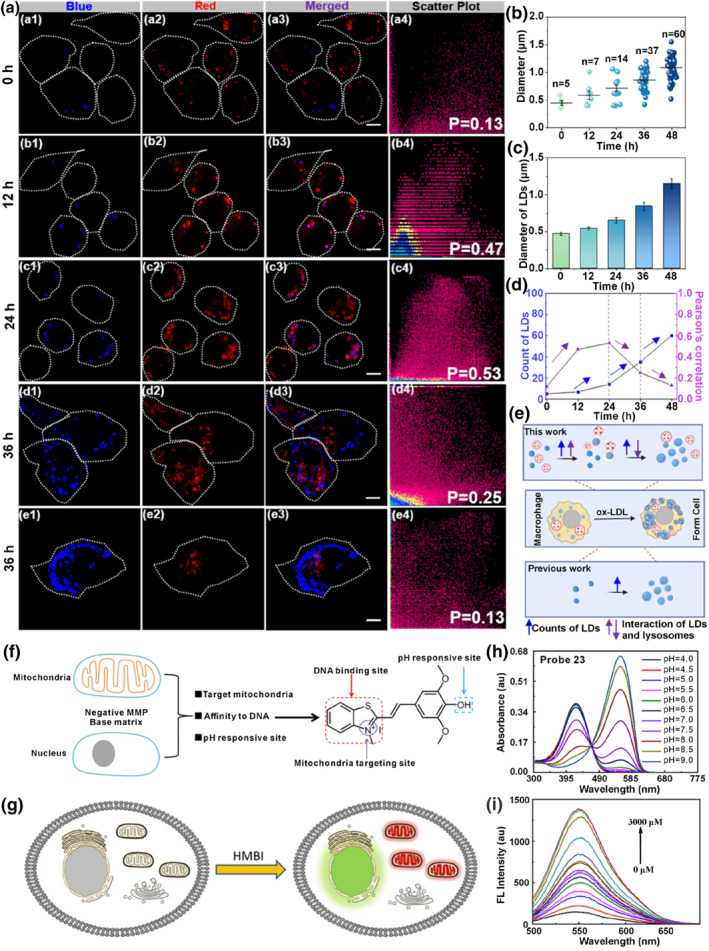
(a) Colocalization images and Pearson correlation coefficient of RAW 264.7 cells labelled with probe **20** after treatment with ox‐LDL for different times. (b) Diameter distribution of LDs of each cell. (c) Diameter of LDs per cell at different times. (d) Changes in the Pearson's colocalization coefficient and number of LDs foam during cell formation. (e) Schematic diagram of probe **20** two‐color visualization of LDs and lysosomes as well as their interactions. (f) Design strategy of probe **23**. (g) Sensing mode of probe **23**. (h) Absorption spectra of probe **23** at different pH values. (i) FL titration spectra of probe **23** with DNA. Reproduced with permission from refs. [^95^, ^98^] Copyright 2021 American Chemical Society. Copyright 2024 American Chemical Society.

In 2024, Kong et al. presented a fluorescent probe **20** to image lysosomes and LDs based on a benzospiropyran‐julolidine derivative, the probe exhibited switchable molecular structures and photophysical properties in diverse pH and polarity environments.^[95]^ In lysosomes, it was protonated and converted to the positively charged ring‐opened form and gave strong red emission (*λ*_em, max_ = 620 nm), while in neutral LDs with small polarity, probe **20** existed in a ring‐closed configuration with blue fluorescence (*λ*_em, max_ = 425 nm). Profiting from the bicolor and dual‐targeting properties, probe **20** served as a formidable instrument to detect atherosclerosis (AS) by simultaneously monitoring the LDs overproduction, the abnormal lysosomal morphology, and the cooperation between LDs and lysosomes (Figure 9a–e).

Recently, Niu and co‐operators fabricated a pH‐sensitive probe **21** to discriminatively visualize LDs and lysosomes.^[96]^ The probe was designed by linking a pH‐responsive quinoline group to a carbazole derivative. It presented in the protonated state in lysosomes (*λ*_em, max_ = 595 nm) to give a red fluorescence, and existed in the neutral form in LDs (*λ*_em, max_ = 445 nm) to give a blue fluorescence. Notably, probe **21** escaped from lysosomes to LDs when lysosomes were alkalized. Lysosomal pH is upregulated during cell apoptosis, and decreased during autophagy, so the probe only labelled LDs in blue emission when cells underwent apoptosis and showed enhanced red emission and almost unchanged blue emission during autophagy. Therefore, probe **21** enabled the discriminative visualization of apoptosis and autophagy via the subcellular localization and emission signals.

In 2024, Wang et al. designed a pH‐sensitive probe **22** to visualize lysosomes and ER by connecting 7‐hydroxy‐coumarin group to a curcumin difluoroboron scaffold.^[97]^ The protonation and deprotonation of 7‐hydroxycoumarin moiety resulted in different optical properties of probe **22**, allowing for visualization of lysosomes and ER in dual emission channels. This probe could be used to reveal the variations of pH in lysosomes (*λ*_em, max_ = 630 nm) and ER (*λ*_em, max_ = 755 nm) under dexamethasone‐induced apoptosis.

Besides lysosomes, the nucleus is also an acidic organelle due to the presence of basophilic DNA and RNA. In 2021, Yu and co‐workers reported the first pH‐sensing molecule **23** to simultaneous dual‐color visualization of nuclei and mitochondria due to the difference in subcellular pH environments (Figure 9f–i).^[98]^ The probe was developed by the rational combination of a weak electronic donor to the benzothiazolium salt that could bind to nucleus DNA. Meanwhile, the hydroxyl group was installed on the hemicyanine skeleton to allow pH responsiveness. The probe had a cationic molecular structure and certain affinity to DNA, which could simultaneously label the mitochondria and nucleus. In living cells, probe **23** existed in a basic form in mitochondria, yielding red emission (*λ*_em, max_ = 605 nm), and was protonated when intercalated into basophilic nucleus DNA to give green fluorescence (*λ*_em, max_ = 550 nm). The authors applied **23** to visualize drug‐induced cell apoptosis in SiHa cells through tracking changes in the two emission signals. Drug stimulation resulted in a dramatic decrease in the red channel in mitochondria, distinct from mitochondria, and the green emission in the nucleus remained basically unchanged during cell apoptosis.

LDs are weakly alkaline electroneutral organelles.^[99]^ By contrast, nucleoli are a faintly acid electronegative organelle.^[100]^ After fully considering the differences in acid‐base environments between the nucleus and LDs, Hu and colleagues prepared a pH‐sensitive reversible probe **24** to target LDs and nucleoli with two emissions in 2023.^[101]^ The pH‐triggered charge reversible fluorescent probe was constructed by linking a cinnamaldehyde group and a benzoindole salt derivative with a *π*‐conjugated system. Based on the pH‐dependent spirocyclization reaction, the positively charged ring‐opening form bound to RNA through hydrogen bonding and electrostatic interaction, which was confirmed by the titrated measurements and molecular docking simulations. Meanwhile, the lipophilic spirocyclization electric neutral form could light up the neutral LD, and the fluorescence shifts from red to blue compared to nucleoli due to the reduction of conjugate degree. Probe **24** has been successfully applied for visualizing the direct physical contact between LDs and nucleolus for the first time. Further exploration of the cooperation between them showed that the number of LDs in the cytoplasm was less susceptible to external stimulation compared with those in the nucleus.

Recently, Shen and co‐workers reported a combinatorial regulation strategy to prepare dual‐targeted single fluorescent probes based on pentacyclic structure. Pentacyclic pyrylium derivatives were considered promising candidates for dual‐labelled probes **25** (**25–1, 25–2, 25–3, 25–4** and **25–5**) due to their diverse cellular uptake behaviour.^[102]^ Typically, the partial aggregation phenomenon of the pentacyclic scaffold allowed it to enter cells via both endocytosis and simple diffusion pathways simultaneously. The endocytosis endowed this structure with inherent lysosomes‐targeting feature. Five different types of dual‐target probes were developed by manipulating a specific organelle‐targeting moiety on the latent dual‐target scaffold. Moreover, deprotonation of the phenolic hydroxyl group led to a red shift in fluorescence emission, and the pH‐triggered dual‐color emission characteristics enabled visualization of lysosomes and other organelles in separate emission channels. These probes exhibited green emission in lysosomes and red emission in others (including mitochondria, nucleolus, LDs and ER). The dual‐labelled lysosomes/mitochondria and lysosomes/LDs probes were used to real‐time visualize the dynamic process of mitophagy and lipophagy by simultaneous dual‐color/dual‐organelle imaging, and experimental results revealed that mitophagy was severely impaired in the Parkinson's disease (PD) cell model. This work provided a promising general strategy for the subsequent development of single‐molecule dual‐labelled probes.

Wu et al. prepared probe **26** to illuminate mitochondria and LDs via the dual‐emission mode based on a hemicyanine‐rhodamine fluorophore.^[103]^ The design concept of dual‐organelle targeting of **26** was according to the reversible spirocyclization reaction mechanism. Its ring‐opening structure exhibited a positive charge that targeted mitochondria and displayed a red emission (*λ*_em, max_ = 580 nm). In contrast, the ring‐closed form with electroneutral and lipophilic properties caused inherent binding to LDs and gave a blue fluorescence (*λ*_em, max_ = 450 nm). The probe could serve as a robust tool to visualize the pH variations and mitochondria‐LDs interactions in living cells via a ratiometric manner.

In addition to targeting dual organelles, probes targeting three organelles with muti‐color have been reported to in‐depth explore the cooperation between organelles. Feng and co‐workers constructed a single‐molecule three‐targeted probe **27**, which was designed based on a coumarin‐merocyanine hybrid scaffold.^[104]^ The reversible cyclization mechanism in the probe achieved a ratiometric response to pH changes with a *p*K_a_ value of 6.68. Under acidic condition, it presented in ring‐opened merocyanine (MC) structure and was transformed into a ring‐closed spiropyran (SP) form in an alkaline environment. These two structural skeletons exhibited great differences in conjugated degree, charges, hydrophilicity/hydrophobicity, and photophysical behaviors. Due to the cationic indolium moiety, the MC form targeted mitochondria through electrostatic attraction and gave a NIR emission (∼700 nm). Meanwhile, the neutral lipophilic SP form could target LDs and yielded a green emission (∼480 nm) of the coumarin unit. Moreover, the weakly basic SP form accumulated in lysosomes and subsequently converted into the MC form due to the acidic microenvironment. Therefore, **27** simultaneously illuminated mitochondria/lysosomes (NIR emission) and LDs (green emission), and mitochondria and lysosomes could be identified through their distinct morphology and fluorescence intensities. The three‐targeting probe **27** was applied for real‐time and simultaneous imaging of the interplays between mitochondria, lysosomes and LDs.

Rhodamine and hemicyanine are the most commonly used pH‐sensitive fluorescent structures for studying organelle interactions. Rhodamine has a spiro structure that can undergo a reversible ring‐closing and ring‐opening process under different acidity levels, providing a turn‐on fluorescence signal changes for proton sensing. Meanwhile, Rhodamine can also cooperate with suitable fluorescent donor to obtain dual‐color pH sensing ability through the FRET mechanism. Since the emission profiles of coumarin and hemicyanine can be well matched with rhodamine absorption, probes **14** and **26** were constructed by connecting rhodamine with coumarin and hemicyanine through appropriate linker. Surprisingly, probes **14** and **26** were simultaneously located in two organelles with different acidity. They were used to monitor organelle interaction in pathological processes since they can distinguish the two organelles by distinct fluorescence signals.

Terminal phenolic hydroxyl, quinoline nitrogen and spiro‐ring are widely utilized in the hemicyanine framework as pH‐responsive sites. Upon acidification, the terminal phenolic hydroxyl is protonated and exhibits strong green emissions. However, significant red‐shifted emission occurs after the phenolic hydroxyl becomes deprotonated under alkaline conditions. Utilizing the pH differences between different organelles, probes **15**, **16** and **23** could discriminative visualization of mitochondria/lysosomes and mitochondria/nucleus in two sets of emission signals. Quinoline nitrogen was used for pH sensing via the protonation/deprotonation function in probe **21**, which was fabricated by attaching a quinoline unit to a carbazole group. The basic and lipophilic properties under acidic and alkaline conditions enable probe **21** to target lysosomes and LDs. The probe appeared in protonated state to yield a red emission in lysosomes, and it existed in the neutral state after deprotonation to give a blue emission in LDs. Similar to rhodamine, the hemicyanine equipped with a spiro‐ring is also a classic photochromic structure whose emission can be adjusted by regulating the acid‐base environment. Benzene ring could be attached to the indole ring to extend the emission wavelength. Probes **19**, **20**, **24** and **27** were developed based on this mechanism. These pH‐dependent spirocyclic structures were present in neutral and lipophilic chemical structures upon base addition, which were expected to label hydrophobic LDs in short wavelength fluorescence. Meanwhile, they showed red fluorescence after transforming into its ring‐open form upon acidification and located in acidic organelles such as the nucleus and lysosomes. Due to this behavior, this kind of skeleton has shown great potential to visualize the interplay between different organelles in two colors.

## 4 ANALYTE CONTROLLED FLUORESCENT PROBES

Intracellular redox dynamic balance is critical to human physiological functions. To maintain the intracellular redox equilibrium, two critical intracellular signalling molecules, reactive sulfur species (RSS) and reactive oxygen species (ROS), can react and transform with each other.[^105^, ^106^] Disruption in the normal ROS or RSS level can eventually lead to disorders in organelle communication and induce a variety of diseases. In addition, enzymes are critical macromolecules in life science and biochemistry. They play essential roles in the catalytic process of biochemical reactions.^[107]^ As a biomarker of most diseases, the activity and expression of enzymes have been proved to be tightly related to a number of human diseases, especially cancer.^[108]^ Therefore, probes for detecting redox substances and enzyme activity can potentially become a useful tool for early clinical diagnosis of diseases.

**FIGURE 10:**
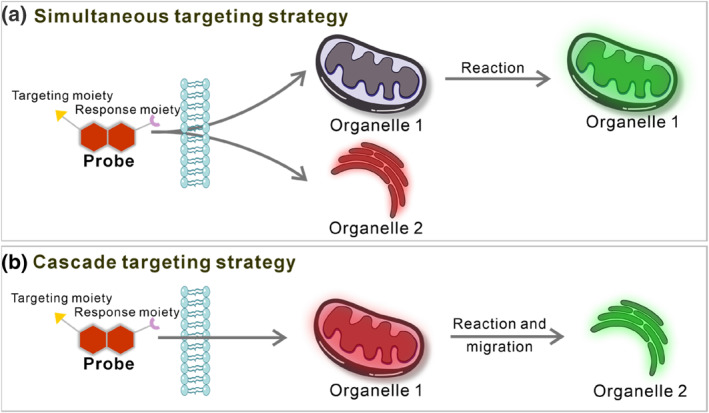
Schematics of simultaneous targeting probes and cascade targeting probes.

Analyte controlled fluorescent probes can be divided into two major classes according to their different entry time into different organelles: simultaneous targeting probes and cascade targeting probes (Figure 10). The simultaneous targeting probes are distributed simultaneously in different organelles after entering the cell. Differences in active species content or microenvironments enable them to emit different colors within these organelles. The cascade targeting probes light up different organelles via migration between organelles, and the whole process is like a “relay race”. They can alter their subcellular localization through variations in the physical microenvironments of organelles or disruption in their molecular skeletons. The decreased affinity of the original organelles for the probe leads to probe release and migration to other organelles, thus realizing their dual‐organelle imaging capability.

### 4.1 Analyte controlled fluorescent probes simultaneously targeting two organelles

**FIGURE 11:**
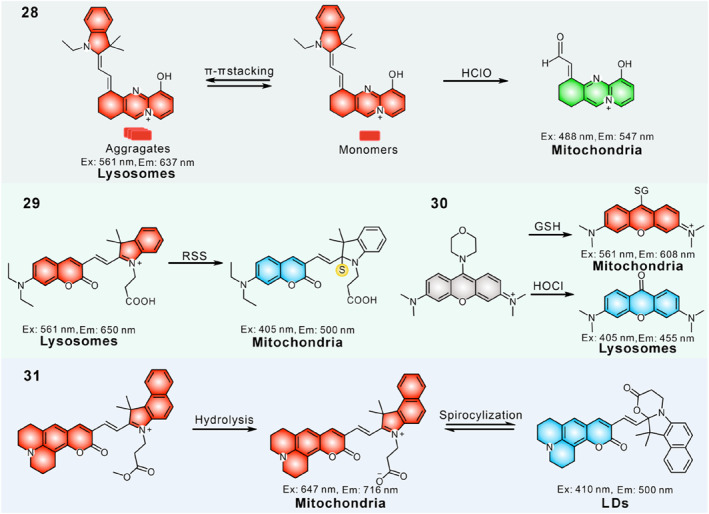
The molecular structures of the analyte controlled fluorescent probes simultaneously targeting two organelles.

The reaction with reactive species provides another feasible approach to prepare single‐molecules to target duple organelles in separate channels. If the analyte controlled fluorescent probe can simultaneously target two organelles and react within one organelle, the goal of dual‐color imaging of organelle interaction can be achieved. These probes are equipped with analyte recognition units and have the targeting ability of two organelles with different levels of analyte content. Once they enter the cell, they will be distributed in the two organelles, and then react with the substance of interest in one organelle, emitting fluorescence different from the other organelle (Figure 11).

**FIGURE 12:**
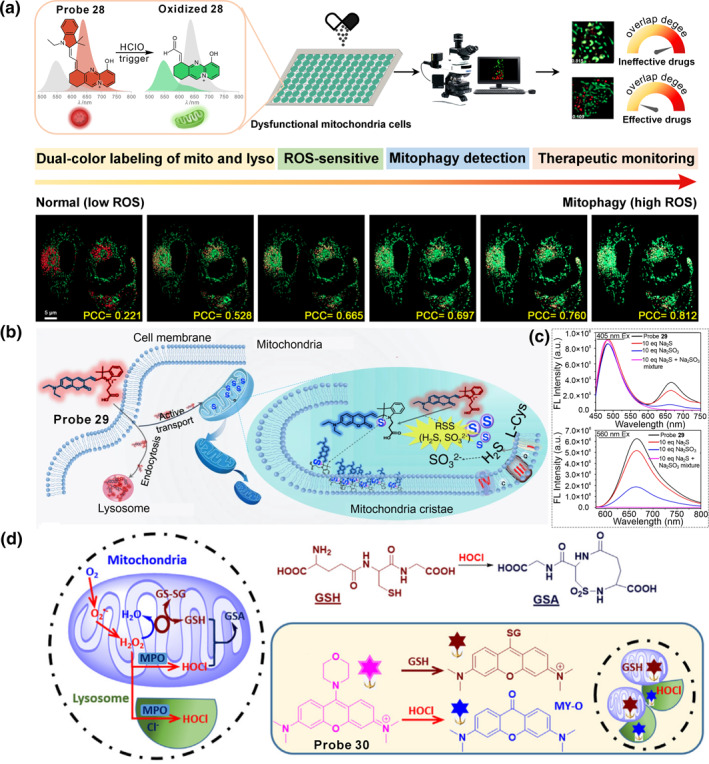
(a) Schematic representation of the possible working mechanism of the probe **28**. (b) Proposed mechanism of probe **29** labelling mitochondria and lysosomes in two‐colors. (c) Fluorescence spectra of probe **29** response toward Na_2_S and Na_2_SO_3_. (d) Design rationale of probe **30**. Reproduced with permission from refs.[^109^, ^110^, ^111^] Copyright 2024 Wiley‐VCH. Copyright 2020 Springer Nature. Copyright 2024 Cell Press.

Recently, our group developed a probe **28** based on a hemicyanine skeleton to simultaneously visualize mitochondria‐lysosome interactions and intracellular oxidative stress (Figure 12a).^[109]^ The near planar structure and multiple intermolecular interactions that drove the aggregation and energy‐dependent endocytosis pathway mediated the red aggregate distribution in lysosomes. Transmembrane electrical potential dominated the free monomer accumulation in mitochondria, and the bond cleavage triggered by basal HClO caused it to give green emission in mitochondria. Information from super‐resolution fluorescent imaging facilitates understanding the dynamics of mitochondria–lysosome interactions and the redox state upon different stimuli as well as the therapeutic effects of the drugs used in regulating mitochondria dysfunction. This work built a bridge between the abnormal mitochondria‐lysosome interaction and mitochondria‐related diseases, providing researchers a valuable tool for disease diagnosis and pharmacodynamic evaluation.

In 2020, our group synthesized a coumarin‐hemicyanine derivative **29** allowing the simultaneous targeting of mitochondria and lysosomes (Figure 12b,c).^[110]^ The positively charged indolenium achieved the mitochondria‐targeting capacity, and the specific *N*‐substituent guaranteed aggregation and delivered into lysosomes via energy ‐ dependent endocytosis. The probe itself emitted red emission (∼650 nm) in lysosomes, which reacted with RSS in mitochondria broke the conjugated systems, generating a new signal in blue emission (∼500 nm). Consequently, probe **29** illuminated lysosomes and mitochondria in red and blue emissions, respectively. Meanwhile, due to the presence of the molecular rotor, **29** could respond to viscosity. Using the probe, the mitochondria‐lysosomes contact (MLC) sites were precisely localized and the local viscosity enhancement during the MLC process was disclosed.

In 2024, Jin's group presented a glutathione (GSH) and hypochlorous acid (HClO) dual‐responsive fluorescent probe **30** based on a 9‐morphorino pyronine dye (Figure 12d).^[111]^ Probe **30** could simultaneously anchor lysosomes and mitochondria due to the co‐existence of the lysosomal‐targeted morpholine moiety and mitochondrial targeted pyronine unit. The probe itself emitted no fluorescence, which reacted with HClO in lysosomes gave a blue emission (*λ*_em, max_ = 455 nm) and reacted with GSH in mitochondria gave a red emission (*λ*_em, max_ = 608 nm). Therefore, lysosomes and mitochondria could be discriminated effectively in separate channels. Subsequently, the mitochondria‐lysosome interaction and GSH‐HClO crosstalk during cell ferroptosis were studied by probe **30**, and revealed that GSH levels decreased under erastin treatment, HClO levels burst under RSL3 treatment, and GSH and HOCl increased simultaneously during FIN56‐ and FINO2‐induced ferroptosis processes.

Very recently, Jiménez‐Sánchez's group reported a dual‐color molecular probe **31** to monitor mitochondria‐LDs dynamics by integrating an ester fragment into a coumarin‐merocyanine hybrid platform.^[112]^ The positively charged indolenium enabled it to localize in mitochondria and gave a red emission. Meanwhile, probe **31** could be hydrolysed by cytosolic esterase and converted into the spirocyclic form with blue‐shift emission; the higher neutral lipophilicity cyclization product drove LDs' permeation with the green‐emission feature. Probe **30** was successfully applied to visualize the dynamic changes during LDs contraction and expansion processes stimulated by pH disorder and autophagy induced by starvation.

### 4.2 Analyte controlled fluorescent probes target two organelles in cascade

The conversion of probes from cationic form to neutral structure in mitochondria can induce their transfer to other organelles. Such probes locate in mitochondria after passing through the cell membrane, where they undergo chemical reactions with redox species or enzymes to generate a new product with different optical performance and localization ability, which can then migrate to other organelles.

**FIGURE 13:**
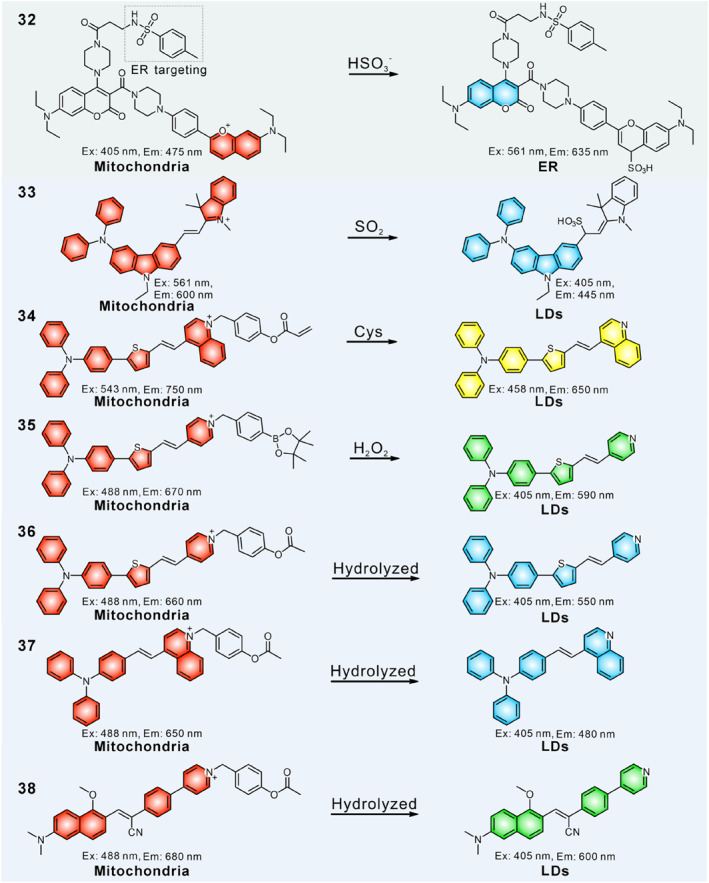
The molecular structures of analyte controlled fluorescent probes cascade to target two organelles.

As shown in Figure 13, some cationic structural dyes could transform into neutral structures after reacting with ROS, RSS and enzyme in mitochondria, and then migrate to ER and lipid droplets. In 2024, Yang's group created a mitochondria and ER dual‐targeted fluorescent probe **32** based on the SO_2_‐driven intramolecular reaction strategy.^[113]^ The probe mainly consisted of a benzopyranium cationic structure, a 7‐diethylaminocoumarin dye, and a methyl sulphonamide group that targeted ER. In their design, the 7‐diethylaminocoumarin dye functioned as the FRET donor, the cationic benzopyronium group not only acted as the FRET acceptor, but also served as a mitochondria‐targeting group and the SO_2_ response site. Under natural conditions, probe **32** possessed a positive charge that embedded in mitochondria via electrostatic attraction, and gave a red emission via the FRET effect. Meanwhile, the cation structure could be disrupted (within 5 s) by SO_2_ in mitochondria and ultimately transformed into a neutral lipophilic structure, leading to migration to the ER and emitting blue fluorescence due to the destroyed FRET effect. Finally, mitochondria and ER were marked in red (*λ*_em, max_ = 635 nm) and blue (*λ*_em, max_ = 475 nm) emission colors, respectively. Given the fluctuations of SO_2_ content during mitochondria‐ER interactions, such a probe allowed for in situ and dynamic visualization of mitochondria‐ER interplay in mitochondrial dysfunctional living cells and zebrafish.

**FIGURE 14:**
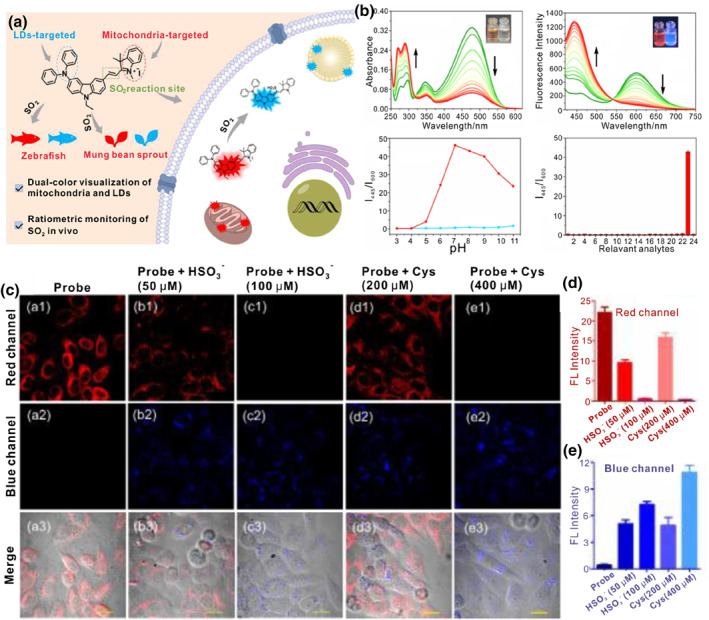
(a) Schematic diagram of the probe **33** to dual‐color target LDs and mitochondria and the mechanism of response to SO_2_. (b) Spectral response of probe **33** toward SO_2_. (c) Fluorescence images of probe **33** detecting exogenous and endogenous SO_2_ in HeLa cells. (d) (e) The quantification of the red and blue channels in the images in (c). Reproduced with permission from ref.^[114]^ Copyright 2023 Elsevier.

In addition, Lin and colleagues demonstrated a ratiometric SO_2_ fluorescent probe **33** to illuminate mitochondria and LDs in two emission colors (Figure 14).^[114]^ The probe was built by the diphenylamine group, indolium and carbazole derivatives. Under normal physiological conditions, the cationic structure with extended *π*‐conjugation enabled probe **33** to selectively accumulate in mitochondria and give a red emission. Meanwhile, probe **33** converted into a neutral lipophilic triphenyl‐like structure after Michael addition reaction in the presence of mitochondrial SO_2_ derivatives, which moved to LDs and emitted strong blue emission. Therefore, mitochondria and LDs were simultaneously labelled in red (*λ*_em, max_ = 600 nm) and blue (*λ*_em, max_ = 445 nm) fluorescence at appropriate levels of SO_2_ exposure. The authors have successfully employed the probe to monitor the interaction between mitochondria and LDs and to track SO_2_ levels in vivo.

In 2023, He and Mao designed the first Cysteine (Cys)‐responsible fluorescent probe **34** for discriminating mitochondria and LDs in dual emission colors.^[115]^ Probe **34** was designed in three parts: the quinolinium moiety targeted the probe into mitochondria, the lipophilic triphenylamine part was used for targeting LD, and the acrylate group served as a specific Cys‐sensitive site. Probe **34** was initially enriched in mitochondria with red fluorescence (*λ*_em, max_ = 750 nm) driven by positively charged quinolinium ions. Subsequently, a reaction occurred between probe **34** and mitochondrial Cys, generating a neutral lipophilic molecule with blue‐shifted emission (*λ*_em, max_ = 650 nm), which escaped from mitochondria and transferred to LDs. With the assistance of probe **34**, the Cys‐triggered two‐emission imaging in mitochondria and LDs during apoptosis under different inducers was clearly visualized through wash‐free staining.

In 2021, Tang et al. fabricated a multifunctional AIE fluorescent probe **35** based on a triphenylamine derivative.^[116]^ A pyridinium cationic moiety and a borate ester segment were introduced as a mitochondria‐targeting group and H_2_O_2_ activatable site, respectively. This probe was initially located in mitochondria and emitted NIR fluorescence (*λ*_em, max_ = 670 nm). Probe **35** partially converted into a yellow‐emissive (*λ*_em, max_ = 590 nm) neutral lipophilic molecule after reaction with mitochondrial H_2_O_2_, which could migrate to LDs. Unlike the aforementioned fluorescent probes, which only applicable to imaging, probe **35** could also generate ROS for photodynamic therapy (PDT).

Some esterase probes can be localized in mitochondria thanks to the negative membrane potential. Their recognition site departs in the presence of mitochondrial esterase, generating a neutral lipophilic structure that can migrate to LDs, and emission enhancement occurs in a short wavelength channel (Figure 14). Therefore, the cell viability can be estimated from the subcellular localization and changes in emission wavelength. In 2022, Dong and co‐workers designed a dual‐organelle targeted AIE probe **36** for evaluating cell viability and killing tumor cells.^[117]^ This compound consists of three sections: a lipophilic triphenylamine fragment, a cationic pyridinium moiety and an esterase hydrolysis site. The probe first enriched in mitochondria with red fluorescence, and lighted up LDs with blue fluorescence after being hydrolysed by the mitochondrial carboxylesterase. This probe has been successfully utilized in evaluating cell viability. More importantly, probe **36** was applied to the efficient PDT of cancer cells due to its high ROS generation capacity under white light irradiation.

**FIGURE 15:**
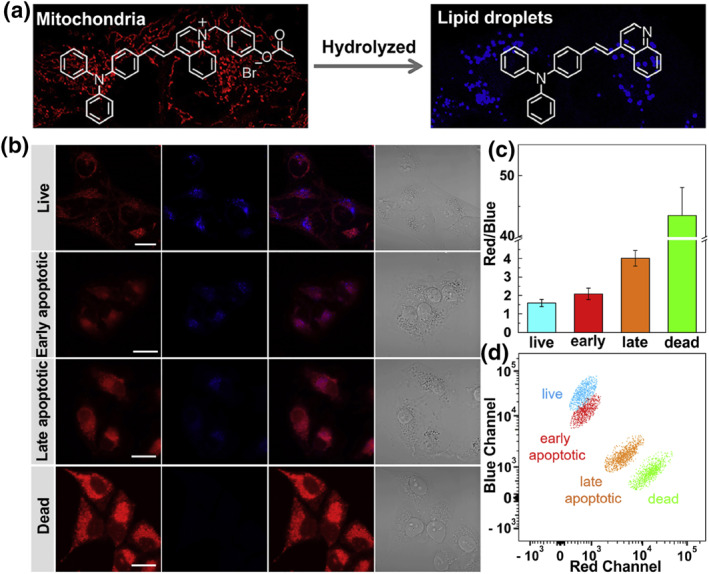
(a) The mechanism of probe **37** to achieve dual emission colors in mitochondria and LDs. (b) Fluorescence images of evaluating cell viability using probe **37**. (c) The relative fluorescence intensity ratio in red and blue channels in images (b). (d) Statistical analysis of cell viability stained with probe **37** by flow cytometry. Reproduced with permission from ref.^[118]^ Copyright 2020 Elsevier.

Tang et al. constructed a triphenylamine derivative‐based probe **37**, which targeted LDs through hydrolysed by mitochondrial esterase, accompanied by a blue‐shift in fluorescence emission (Figure 15).^[118]^ Probe **37** first localized in mitochondria and gave a red fluorescence. Then, the carboxylesterase‐mediated hydrolysis converted probe **37** into a neutral product which moved to LDs and released blue emission. Therefore, probe **37** could emit red and blue emission to illuminate mitochondria and LDs, respectively. Since the fact that esterase activity could indicate cell viability, probe **37** was successfully applied for evaluating cell viability. Of particular interest was that probe **37** could also selectively differentiate different cell physiological stages through flow cytometry and confocal microscopy. In 2023, Fan and co‐workers developed probe **38** that enabled dual visualization of mitochondria and LDs based on the same recognition enzyme.^[119]^ The design concept and responding performance of probe **38** were similar to the above probes **36** and **37**; it could light up mitochondria and LDs in red (*λ*_em, max_ = 680 nm) and green emissions (*λ*_em, max_ = 600 nm) and could be used for cell viability assessment.

The framework structures of most reported cascade targeting fluorescent probes mainly include pyridine or quinoline salt derivatives with triphenylamine skeleton. In the presence of reactive substances, their recognition site departs, and the fluorescence undergoes a blue shift due to the changes in electron‐absorbing capacity. In addition, the transition to a neutral structure causes them to lose their electrostatic attraction with mitochondria and thus migrate to LDs. Although their main luminescent group is similar, they can be triggered by different analytes through modification with different response groups.

## 5 ORGANELLE STATUS CONTROLLED FLUORESCENT PROBES

In general, mitochondria can absorb cationic structures due to their negative MMP; molecules carrying weakly alkaline amines are preferentially enriched in lysosomes due to the weak acidic lysosomal microenvironment. Therefore, the weakening of electrostatic forces between mitochondria and probes or lysosomal alkalization could reduce their specific targeting ability. Mitochondrial depolarization and lysosome pH enhancement occur in the process of cell death or under other stimuli, the dyes accumulated in the mitochondria and lysosomes will move to other region, bring in secondary targeting. Meanwhile, fluorescence colors can concurrently switch due to the variations in the microenvironments.

### 5.1 Migration dependent on mitochondrial membrane potential depolarization

**FIGURE 16:**
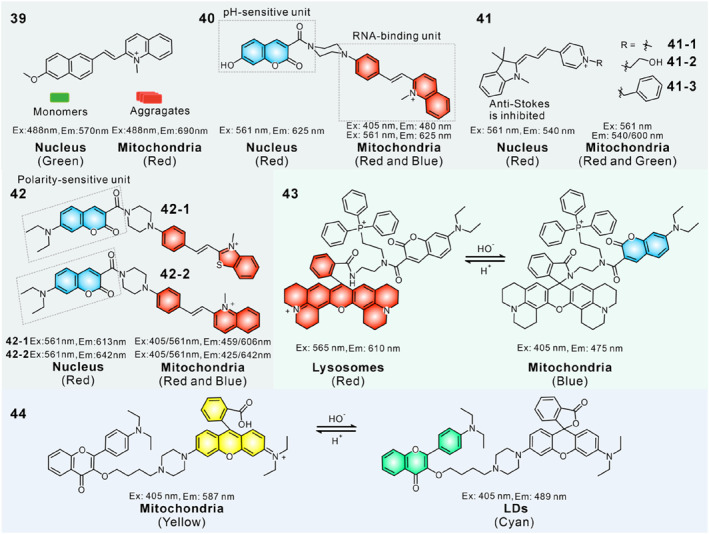
The molecular structures of fluorescent probes achieve dual‐targeting dependent on membrane potential depolarization‐induced subcellular immigration.

Positively charged fluorescent dyes with good membrane permeability can accumulate in mitochondria via electrostatic force, and there is a positive correlation between their content in mitochondria and the ΔΨ_m_. During the cell death process, the MMP disappears gradually, and the molecules localized in the mitochondria will move to other regions of the cell, leading to the secondary targeting. Based on this design principle, these probes could realize the migration between two organelles, thereby achieving the purpose of dual‐color discrimination of cell status (Figure 16).

In 2018, Lin's group reported a mitochondria and nucleus migration fluorescent probe **39** to visualize the change of MMP in a dual‐color mode. Probe **39** was composed of a weak electronic donor methoxynaphthalene unit and a positively charged quinolinium salt.^[120]^ It was enriched in mitochondria in healthy cells due to the strong electrostatic attraction between its cationic salt and high negative MMP, and the high concentration in mitochondria led to the formation of aggregates with deep‐red fluorescence. Meanwhile, the probe possessed certain affinity to DNA due to its structure resembling Hoechst 33342, and could escape from mitochondria and immobilize into nuclear DNA to form monomers with green emission after the reduction of MMP in unhealthy status. In addition, probe **39** reversibly moved back to mitochondria and emitted deep‐red fluorescence as ΔΨ_m_ recovered. Thus, the reversible migration between mitochondria and nucleus and the corresponding emission color changes of probe **39** could serve as indicators for evaluating cell viability.

**FIGURE 17:**
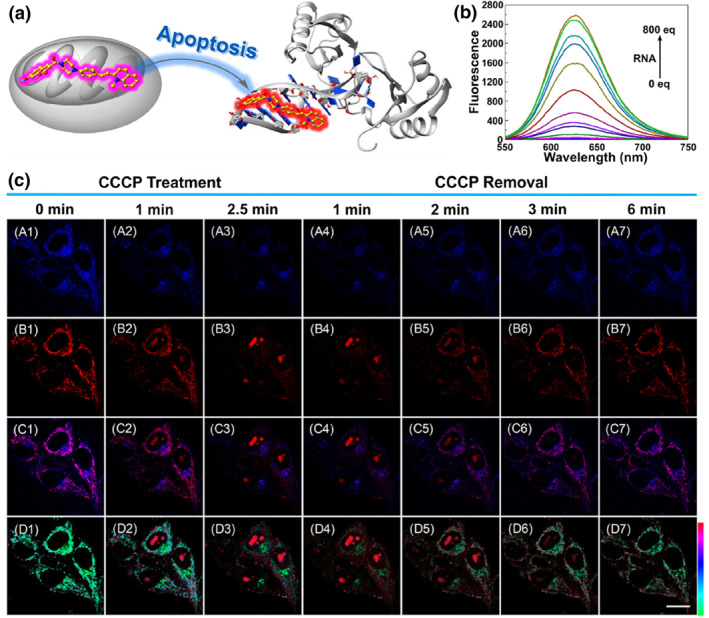
(a) Schematic diagram of probe **40** to dual‐color targeting mitochondria and nucleus. (b) Fluorescence spectrum of probe **40** in the presence of RNA. (c) Fluorescence and ratiometric images of probe **40** in response to ΔΨ_m_ changes in HepG2 cells. Reproduced with permission from ref.^[121]^ Copyright 2019 American Chemical Society.

In 2019, Lin's team further developed a pH‐sensitive fluorescent probe **40** for monitoring the MMP changes in dual‐color mode (Figure 17).^[121]^ This molecule consisted of three parts: a blue‐emissive 7‐hydroxyl coumarin dye as pH recognition site and electron donor, a red‐emissive quinolinium salt derivative as RNA binding unit and electron acceptor, and a piperazidine group as a bridge for the two parts. In healthy cells, this probe localized in mitochondria with basic matrixes and displayed both red fluorescence from the cationic quinolinium salt fluorophore and blue fluorescence from coumarin fluorophore. Upon the depolarization of mitochondria, part of probe **40** would leave mitochondria and bound to the RNA grooves. Due to the basophilla character of RNA, the hydroxyl‐coumarin dye was protonated and the blue emission faded. Thus, probe **40** could light up mitochondria in both red and blue fluorescence, while the nucleus showed only red emission. The reversible fluorescence change depended on ΔΨ_m_, which significantly decreased during cell apoptosis. Probe **40** enabled the real‐time ratiometric visualization of cell apoptosis under different stimuli.

In 2022, Tian's group developed three upconversion fluorescent probes **41** (**41‐1**, **41‐2** and **41‐3**) to image mitochondria and nuclei with dual‐color in living cells.^[122]^ These probes were constructed with an electron‐donating indole group and different electron‐withdrawing pyridinium units. Methyl and ethoxy units were chosen as the sidechains to obtain high affinity to RNA, and a large phenmethyl group was selected as a control group. In healthy living cells, the probe labelled mitochondria with normal MMP in both green (anti‐Stokes) and red emission (Stokes). After the decrease of MMP, the probes detached from mitochondria and migrated into nucleolus owing to the binding affinity to RNA, and gave only a red emission because the anti‐Stokes upconversion emission was efficiently inhibited when combined with RNA. As a result, these target‐switchable probes could detect the reversible ΔΨ_m_ changes in a dual‐color mode in cells and tissues.

In 2023, Tian's group designed two target‐switchable fluorescent probes **42** (**42–1** and **42–2**) to detect ΔΨ_m_ according to the polarity discrepancy in mitochondrial inner membrane and RNA.^[123]^ Probes **42–1** and **42–2** were constructed by connecting a polarity‐responsive fluorophore coumarin to a red‐emissive benzothiazole salt derivative or a quinolinium salt group via a piperazidine group, which showed both red and blue emission in low polarity environments and only showed red emission when polarity was elevated. Meanwhile, the cationic structure exhibited certain electrostatic interactions with the negatively charged mitochondria and nucleus. Consequently, in cells with normal MMP, probe **42–1** mainly located in mitochondria and gave both red and blue fluorescence attributed to the low mitochondrial polarity. Then partial probe molecules migrated from mitochondria to RNA once ΔΨ_m_ decreased, emitting decreased blue fluorescence and enhanced red fluorescence due to the higher polarity of the nucleus compared to mitochondria. Based on these properties, probe **42–1** was capable of visualizing ΔΨ_m_ changes by emission color changes and staining position.

In 2017, Han et al. reported a target‐switchable fluorescent probe **43** for detecting the depolarization of ΔΨ_m_ and selected lysosomes as the second target.^[124]^ This probe mainly consisted of three parts: a triphenylphosphonium group for targeting mitochondria, a rhodamine‐lactams for responding to pH fluctuations and targeting lysosomes, and an ‘‘always‐on’’ blue‐emissive neutral coumarin fluorophore. The pH‐sensitive probe showed red fluorescence in an acidic environment for blue fluorescence in basic conditions due to the reversible intramolecular cyclization reaction of rhodamine dye. Under normal physiological conditions, the probe localized in mitochondria under alkaline conditions and displayed blue fluorescence from the coumarin dye. When mitochondria lost their high ΔΨ_m_, partial molecules relocated at lysosomes with low pH values and emitted red fluorescence from the ring‐opening rhodamine dye. Therefore, lysosomes and mitochondria were labelled in red and blue fluorescence, respectively. The probe was capable of detecting mitochondrial depolarization in different physiological and pathological processes in a two‐color pattern.

Based on a similar mechanism, Lin's group reported a dual‐color fluorescent probe **44** for monitoring ΔΨ_m_ variations via the migration between mitochondria and LDs.^[125]^ Rhodamine and flavone were selected as an energy acceptor and an energy donor. Its physical properties could be altered through the reversible intramolecular reaction of rhodamine triggered by pH. The cationic ring‐opened form of probe distributed in mitochondria in healthy cells with normal MMP, and emitting yellow fluorescence due to the FRET effect. With the depolarization of ΔΨ_m_, partial probe molecules were released from mitochondria and transformed into the ring‐closed rhodamine form in a low pH environment; this neutral lipophilic structure could target LDs and emit cyan fluorescence from flavonoid dyes. Thus, the subcellular localization and emission colors were switched concurrently. Because ΔΨ_m_ is significantly dependent on cell viability, the probe could visualize cell viability by the treatments of lipopolysaccharide, UV‐exposure and H_2_O_2_.

### 5.2 Migration dependent on lysosomal alkalization

**FIGURE 18:**
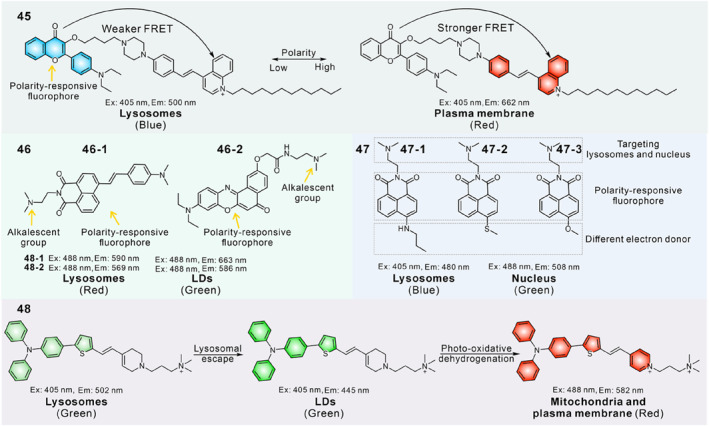
The molecular structures of fluorescent probes achieve dual‐targeting dependent on lysosomal alkalization‐induced subcellular immigration.

Lysosomes require acidic lumen for effective digestion of extracellular material and intracellular macromolecules. Dysregulation of the lysosomal membrane proton gradient impairs the clearance of substrates and ultimately leads to cell disorders. In recent years, a single‐probe that could change its subcellular localization and emission wavelength has been developed to estimate the degree of lysosomal damage (Figure 18). Hydrolases and protons will release once lysosomes are destroyed, causing the weak alkaline probe lose its targeting ability towards lysosomes and transfer to another organelle, and the fluorescence signal changes with variations in microenvironments.

**FIGURE 19:**
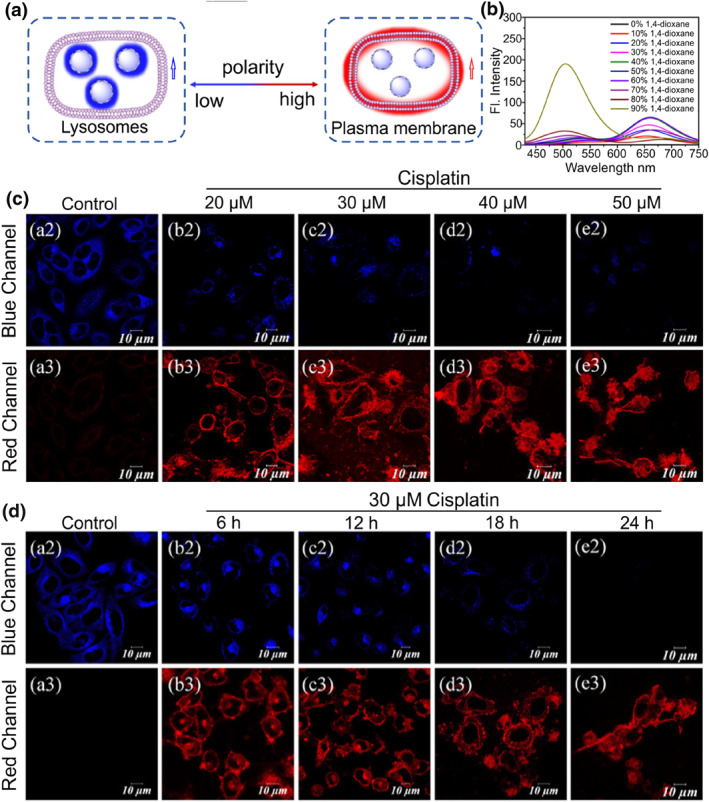
(a) The design principle of probe **45** for visualization of lysosomes and plasma membrane in distinct emission colors. (b) Fluorescence spectrum of probe **45** in the percentage of H_2_O and 1,4‐dioxane. (c) Imaging of HeLa cells pre‐treated with various concentrations of cisplatin for 24 h and subsequently labelled with probe **45**. (d) Images of HeLa cells pre‐treated with 30 μM cisplatin at different times followed by imaging with probe **45**. Reproduced with permission from ref.^[126]^ Copyright 2022 American Chemical Society.

In 2022, Lin's group reported a dual‐target fluorescent probe **45** to detect the drug‐induced cell apoptosis process in a dual‐color mode based on the FRET mechanism.^[126]^ A flavone derivative (*λ*_em, max_ = 400−510 nm) and a cationic quinoline dye (*λ*_em, max_ ≈ 540 nm) were selected as the donor and acceptor, respectively. The flavone dyes with D−A molecular structure were sensitive to polarity changes. In low polarity, the FRET efficiency was weak owing to the mismatch between the emission spectrum of flavonoids and the absorption spectrum of quinoline dye, and gave a blue emission from the flavonoid. In addition, probe **45** emitted red fluorescence of quinoline derivative in high polarity because the degree of FRET efficiency enhanced with the red‐shift emission of the flavone fluorophore. In living cells, this probe is specifically located in lysosomes and displayed in blue emission. Once the cells were treated with cisplatin to induce apoptosis, probe **45** could rapidly leave the lysosomes and light up the plasma membrane with red fluorescence (Figure 19). Thus, the probe could detect cell viability through both the subcellular localization and emission colors.

In 2023, Tian and co‐workers fabricated fluorescent probe **46‐1** to distinguish healthy, early apoptotic, and late apoptotic cells through the migration between lysosomes and LDs.^[127]^ Probes **46–1** and **46–2** were designed by introducing an amine unit to the polarity‐sensitive fluorophores (phthalimide and Nile red). In living cells, probe **46–1** presented in protonated status and bound to the lysosomal membranes with high polarity. In early apoptotic cells, most probe molecules experienced deprotonation and transformed into neutral form due to the increase in lysosomal pH, which exhibited enhanced hydrophobicity and distributed in the inner region with modest polarity. After lysosomal membrane permeabilization during the late apoptotic process, probe **46–1** was released from lysosomes to LDs with ultralow polarity. Meanwhile, the probe displayed red‐shifted emission with increasing environmental polarity, which enabled probe **46–1** to directly recognize the three different apoptotic stages using three different emission signals.

Recently, Tian's group further reported a target‐switchable fluorescent probe **47–2** to visualize cell apoptosis.^[128]^ The polarity‐sensitive probe consists of a naphthylimide dye and a weak basic *N*, *N*‐dimethylethylenediamine group; three different groups were introduced to modulate the sensitivity to environmental polarity. Probe **47–2** displayed apparently higher sensitivity to polarity than probe **47–1** and **47–3**. Probe **47–2** selectively targeted lysosomes with higher polarity; after lysosomal alkalization, it could migrate from the alkalinized lysosomes to the nucleus. Since the lower polarity microenvironment in lysosomes compared to the nucleus, and the emission spectrum of probe **47–2** could reflect the environmental polarity changes, this polarity‐responsive probe could distinguish lysosomes and nucleus in blue and green colors. The probe has been successfully applied in visualizing cell apoptosis by changes in organelle morphology and emission colors induced by different stimuli including UV irradiation, oxidative stress, rotenone, paclitaxel and colchicine.

In 2022, Wang and co‐workers developed a three‐targeted fluorescent probe **48** with organelle transfer capability based on the photoactivatable tandem organelle imaging strategy.^[129]^ The photoactivatable probe was designed based on a triphenylamine derivative. Probe **48** exhibited high polarity sensitivity which was attributed to the ICT effect. After probe **48** entered the cell, it selectively localized in lysosomes initially, displaying weak green emission. When exposed to white light, the probe would release from lysosomes owing to the ROS induced lysosomal damage. Subsequently, one part of the probe enriched in LDs with intense green emission, while the other part experienced the photo‐oxidative dehydrogenation reaction to generate a red‐emissive compound, and accumulated in the mitochondria and cytomembrane. Probe **48** was successfully utilized to long‐term observe the interplay between mitochondria and LDs under the ROS‐triggered oxidative stress.

## 6 COMPOUND STATUS CONTROLLED FLUORESCENT PROBES

**FIGURE 20:**
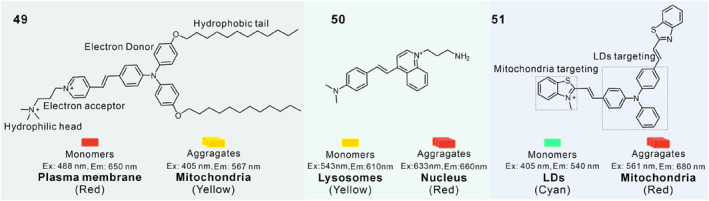
The molecular structures of dual‐targeted fluorescent probes based on the aggregation/monomer strategy.

Aggregation‐induced emission (AIE) has attracted considerable interest in the development of functional dyes owing to their excellent luminescence properties in aggregate states.^[130]^ If AIE derivatives could simultaneously label two organelles and exist in the form of aggregates and monomers, respectively, dual‐color imaging distinguishing dual organelles could be achieved because aggregates and monomers of some AIE luminogens possess different emission colors (Figure 20).

Based on this principle, Tong et al. developed an amphiphilic and cationic AIE sensor **49** to image mitochondria and cytoplasm membrane in a simultaneous and selective manner. One quaternary ammonium salt chain and two C_12_ alkyl chains were introduced to the triphenylamine scaffold‐based probe. Suitable amphiphilic and positively charged structure allowed probe **49** to be simultaneously localized in the cell membrane and mitochondria.^[131]^ Meanwhile, the combined AIE and TICT effects rendered it capable of emitting in two channels in response to different organelle microenvironments. In the plasma membrane, the probe existed in monomers and gave a red emission (*λ*_em, max_ = 650 nm). In mitochondria, molecular aggregates formed due to the aggregation‐induced restriction of intramolecular rotation effect, giving a blue shift emission in the yellow channel (*λ*_em, max_ = 567 nm). Based on the results, probe **49** was successfully utilized to visualize and discriminate the dynamic interactions between cell membrane and mitochondria during cell ROS‐induced apoptosis and cytotoxins‐induced necrosis.

**FIGURE 21:**
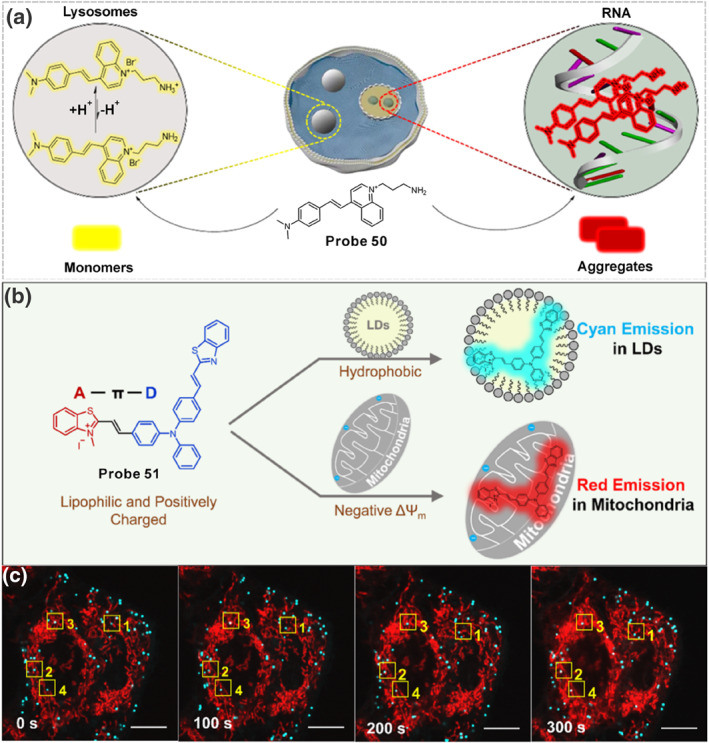
(a) Chemical structure of probe **50** and proposed mechanism to target RNA and Lysosomes in yellow and red colors. (b) Chemical structure of probe **51** and its possible mechanism of dual‐organelle targeting. (c) Time‐lapse imaging of probe **51** in the dynamic interaction between LD and mitochondria. Reproduced with permission from refs.[^132^, ^133^] Copyright 2022 American Chemical Society.

In 2022, Yu et al. reported a versatile single fluorescent probe **50** to image RNA and lysosomes based on the aggregation/monomer principle.^[132]^ The probe was designed by simultaneously introducing a cationic and weakly basic unit to a hemicyanine derivative. In general, the positively charged organic molecules displayed high affinity to negatively charged RNA grooves, and weakly basic fluorophores tend to embed into lysosomes with an acidic microenvironment. Therefore, probe **50** could simultaneously target lysosomes and RNA, and stained lysosomes in yellow color and displayed deep red emission in RNA due to the formation of aggregates. Lysosomal pH increased during apoptosis and decreased during autophagy. Due to the opposite trend of pH changes in lysosomes during these two processes and the probe migrated between RNA and lysosomes as pH varied, apoptosis and autophagy could be differentiated and visualized via the probe localization and emission color (Figure 21a).

Probe **51** was designed by integrating the dual mechanisms of AIE and TICT.^[133]^ The probe aggregated in the aqueous phase due to its hydrophobic properties resulting in increased fluorescence (Figure 21b,c). While it existed in monomer form under hydrophobic condition, the emission peak would be blue‐shifted owing to the TICT effect. Moreover, plus its lipophilicity and cationic properties, the probe was capable of simultaneous labelling of LDs (*λ*_em, max_ = 540 nm) and mitochondria (*λ*_em, max_ = 680 nm) in dual‐emission colors. The LDs‐mitochondria interactions were characterized in cells and in C. *elegans*, showing that LDs played a role in mitochondrial dynamics because some mitochondria underwent fission and fusion upon contact with LDs. Subsequent experiments demonstrated a significantly higher level of contact between LDs and mitochondria appeared in ferroptotic cells. This is the first time that LDs‐mitochondria interactions could be tracked in vivo using a probe.

## 7 SUMMARY AND PERSPECTIVE

The ubiquitous nature of organelle interactions and their significant roles in cellular life activities make them an essential research topic which stimulates the development of dual‐color dual‐target fluorescent probes. The probes capable of dual‐color differentiation of different organelles have shown great potential to elucidate the organelle interaction networks and facilitate a better understanding of biophysical parameters such as cell health and diseased states, mitochondrial membrane potential and lysosomal membrane permeabilization. In addition, anomalous interactions often occur before the disease manifests prominently, so such fluorescent sensors will contribute to the early diagnosis of diseases and timely intervention and treatment. In this review, we provide a systematic overview of the latest progress in the design and construction of these special fluorescent probes for studying organelle communication networks.

According to the differences in microenvironment and active species content in diverse organelles, many dual‐color and dual‐target probes have been developed through rational molecular design. Among them, microenvironment controlled fluorescent probes are the most widely investigated and developed relatively mature. One benefit of developing microenvironment‐based probes for targeting two organelles is low cytotoxicity. Unlike those probes reacting with active molecules in cells, they are able to perform dual‐color imaging with minimal influence on the normal function of cells. In addition, these probes respond to different microenvironments in a real‐time and reversible manner. Previous works have established that abnormal microenvironment fluctuations often occur in stressed cells, and real‐time tracking the dynamic changes in the microenvironment during organelle interactions can provide us with deeper insights into the important biological events. However, these probes also revealed some shortcomings and still have room for improvement. For example, some organelles other than LDs have slight differences in water contents and polarities. This environmental similarity limits the broad application of such probes in complex cellular environment. Besides, some polarity‐sensitive probes suffer from small fluorescence shifts due to weak ICT effects, leading to fluorescent signal acquisition that could not avoid interference between two types of fluorescence signals. This motivates us to strategically maximize electron donor−acceptor interactions through rational design and thus reduce spectral overlap under different polarities. Compared to the widespread utilization of microenvironment‐sensitive probes for studying the organelle interaction, the development of analyte controlled dual‐targeting probes are relatively limited. Several challenges remain to be tackled by chemists when designing suitable reaction‐based single‐molecule used for staining two organelles. (1) The first challenge lies in creating probes that have high sensitivity and rapid response capability, such probes should show ratiometric optical response in basal intracellular reactive substances contents. (2) Improved specificity of identification for preventing false positive signals caused by other cellular active molecules. (3) There are few reports on scaffolds used for cascade targeting strategies, pyridine and quinoline salt derivatives are commonly used. The transition from cationic structure to neutral lipophilic structure after reacting with analyte in mitochondria renders them suitable for cascade targeting of mitochondria and LDs. Particular attention should also be paid to developing reaction‐based probes suitable for imaging other organelles besides mitochondria and LDs.

Despite some progress has been achieved, there are many unmet challenges along with opportunities that still remain in this field. (1) At present, the mechanism of simultaneously targeting dual organelles in distinct channels remains elusive. Therefore, there are still many difficulties in using a single fluorescent probe to achieve differential labelling of organelles. Due to their distinct structural characteristics, targeting of LDs, mitochondria and lysosomes related interaction networks is relatively mature. However, research on other organelle related interaction networks, including the Golgi apparatus, microtubules, and peroxisome, has not yet been investigated using single probes. In addition, compared to fluorescent probes targeting dual organelles, probes with multiple organelles targeting capability are still scarce. Therefore, more probes still need to be developed to realize the clear and separate visualization of organelles with no spectral crosstalk. (2) Except for bioimaging capability, the application of most existing fluorescent probes in subsequent treatment is relatively underexplored. If a single molecule can be designed as a diagnostic‐therapeutic integration platform, the synergistic strategy will avoid the treatment delays and reduce disease recurrence rates during the continuous follow‐up processes. (3) spatial resolution in conventional fluorescent confocal microscopy is plagued by optical diffraction which restricts its ability to observe the fine internal ultrastructure smaller than 200 nm, and the dynamic interactions between organelles are described in a relatively rough pattern. Super‐resolution microscopy surpasses the resolution limit and allows researchers to obtain nano‐scale information concerning organelles in living cells.[^46^, ^134^, ^135^] Therefore, we need to improve the photostability and fluorescence intensity of probes to avoid photobleaching, as the excitation light of the super‐resolution microscope is very strong. In a word, developing single probes with multiple subcellular locations and multiple colors is expected to be a promising future direction. The current achievements are just a small step in this area, so we hope more high‐performance smart molecules will be fabricated to better investigate organelle interaction networks, which is of great importance in biological research and medical diagnosis.

## CONFLICT OF INTEREST STATEMENT

The authors declare no conflicts of interest.

## Data Availability

Data sharing not applicable—no new data generated or the article describes entirely theoretical research.
